# New Insights Into the Evolutionary History of Melatonin Receptors in Vertebrates, With Particular Focus on Teleosts

**DOI:** 10.3389/fendo.2020.538196

**Published:** 2020-09-24

**Authors:** Gersende Maugars, Rasoul Nourizadeh-Lillabadi, Finn-Arne Weltzien

**Affiliations:** Physiology Unit, Faculty of Veterinary Medicine, Norwegian University of Life Sciences, Oslo, Norway

**Keywords:** melatonin receptors, gene duplication, vertebrates, teleosts, medaka, phylogeny, synteny, functional evolution

## Abstract

In order to improve our understanding of melatonin signaling, we have reviewed and revised the evolutionary history of melatonin receptor genes (*mtnr*) in vertebrates. All gnathostome *mtnr genes* have a conserved gene organization with two exons, except for *mtnr1b* paralogs of some teleosts that show intron gains. Phylogeny and synteny analyses demonstrate the presence of four *mtnr* subtypes, MTNR1A, MTNR1B, MTNR1C, MTNR1D that arose from duplication of an ancestral *mtnr* during the vertebrate tetraploidizations (1R and 2R). In tetrapods, *mtnr1d* was lost, independently, in mammals, in archosaurs and in caecilian amphibians. All four *mtnr* subtypes were found in two non-teleost actinopterygian species, the spotted gar and the reedfish. As a result of teleost tetraploidization (3R), up to seven functional *mtnr* genes could be identified in teleosts. Conservation of the *mtnr* 3R-duplicated paralogs differs among the teleost lineages. Synteny analysis showed that the *mtnr1d* was conserved as a singleton in all teleosts resulting from an early loss after tetraploidization of one of the teleost 3R and salmonid 4R paralogs. Several teleosts including the eels and the piranha have conserved both 3R-paralogs of *mtnr1a, mtnr1b*, and *mtnr1c*. Loss of one of the 3R-paralogs depends on the lineage: *mtnr1ca* was lost in euteleosts whereas *mtnr1cb* was lost in osteoglossomorphs and several ostariophysians including the zebrafish. We investigated the tissue distribution of *mtnr* expression in a large range of tissues in medaka. The medaka has conserved the four vertebrate paralogs, and these are expressed in brain and retina, and, differentially, in peripheral tissues. Photoperiod affects *mtnr* expression levels in a gene-specific and tissue-specific manner. This study provides new insights into the repertoire diversification and functional evolution of the *mtnr* gene family in vertebrates.

## Introduction

Melatonin is a highly conserved neurohormone that relays the daily and seasonal variations in photoperiod to an organism. Produced in the pineal gland and retina during the night, melatonin regulates behavior, reproduction, and growth (1, 2). Melatonin is an indoleamine synthesized from the tryptophan/serotonin pathway. In vertebrates, the synthesis of melatonin is controlled by the circadian rhythm in arylalkylamine N-acetyltransferase (AANAT) activity.

Melatonin exerts its actions by binding to specific receptors that belong to the superfamily of G protein-coupled receptors (3–5). The melatonin receptors are members of the α-group of rhodopsin-like receptors. Three melatonin receptor (MTNR) subtypes have been characterized in vertebrates: MTNR1A (MT1, Mel1a), MTNR1B (MT2, Mel1b), and MTNR1C (Mel1c). The latter includes the mammalian GPR50 that has lost the capacity to respond to melatonin (6). Recently, it was shown that the additional teleost *mtnr1a-like* gene is also present in some tetrapods (*mtnr1a2* or *mtnr1a1*. *4*) and consequently constitutes a fourth MTNR subtype named MTNR1D or MTNR1A-like (7–12). The four MTNR monophyletic groups presumably derive from the two rounds of vertebrate tetraploidization early in vertebrate evolution (1R and 2R) (10–12). Sakai et al. reported that teleost species possess the four vertebrate MTNR subtypes but did not observe any further expansion of the *mtnr* gene repertoire in teleosts (11). In contrast, analysis of the Atlantic salmon (*Salmo salar*) genome has revealed the presence of five functional *mtnr* genes and three *mtnr* pseudogenes resulting from duplication of the four vertebrate *mtnr* genes during the teleost-specific 3R and the salmonid-specific 4R tetraploidization events (10, 12).

The four subtypes are expressed in the brain, as well as in peripheral tissues (3–5, 7–9, 11, 13–15). In vertebrates, *mtnr* genes show diurnal variation in expression in brain and retina. Regulation of *mtnr* expression contributes to the overall regulation of melatonin signal transduction. Taking advantage of the recent publication of new genomes, here we investigate the evolutionary history of melatonin receptors in the main vertebrate taxa, with a special emphasis on the impact of the teleost-specific tetraploidization (3R) on their *mtnr* gene repertoire. Our study includes the characterization of new melatonin receptor genes from several recently sequenced vertebrate genomes. As they typically differ in their retention and loss of paralogs derived from the 3R (16), we have included the three sister groups of teleost species. In order to gain a better understanding of the functional evolution of melatonin receptor subtypes, we investigated the tissue distribution of *mtnr* paralogs during the day/night cycle in the medaka (*Oryzias latipes*), a widely used model organism, and using the PhyloFish database (17).

## Materials and Methods

### Gene and Protein Nomenclature

Nomenclature follows the HGNC (mammals), Xenbase (amphibians), and ZFin (teleosts) conventions. For the non-model species, the gene symbols are in lowercase italics and protein symbols in uppercase non-italics. Duplicate receptor genes arising from the teleost tetraploidization (3R) are distinguished by the addition of the suffixes a or b after the gene symbol, whereas those arising from the cyprinid or salmonid tetraploidization (4R) were annotated with the suffixes α and β [according to the annotation previously used in the functional study of the Atlantic salmon *mtnr* (10)]. MTNR (in upper-case letters) refers to the receptor type.

### Identification of Melatonin Receptor Paralogs

Different vertebrate taxa (*n* = 70), selected according to their phylogenetic position and genome availability, were screened for melatonin receptor genes (18–21). Gene sequences were retrieved from genomic assemblies, either using GenBank gene prediction, or by an exhaustive Blast search against GenBank, Ensembl and UCSC genome databases, and GenBank, UCSC and PhyloFish transcriptome databases (17). We used genome assemblies to search for non-annotated *mtnr* genes or to confirm gene loss. Gene coding sequences (CDS) were manually annotated or curated by comparison with well-known orthologous gene sequences according to the canonical donor-acceptor splicing site rule using CLC Main Workbench (QIAGEN). Sequence references and annotations are provided in Supplementary Table 1. Receptor membrane topologies were predicted using the browser TOPCONS browser (22).

### Phylogenetic Analysis

Multiple alignments of melatonin receptor gene sequences were performed using the “accurate alignment” algorithm of CLC Main Workbench, and further manually edited to correct for obvious misalignments and improve sequence alignment. Preliminary phylogenetic trees were built to control the quality of the *mtnr* dataset per species. Phylogenetic trees were inferred from the full amino acid or nucleotide sequences of *mtnr* using either the entire *mtnr* repertoire of vertebrate representatives, or for each *mtnr* subtype, including additional species. Tree topology was inferred with PhyML3.0, using the AIC substitution model selection using the ATGC browser (23, 24). Branch node strength was evaluated by bootstrapping using 100 replicates. Melatonin receptor trees for gnathostome species were not rooted. An additional tree including the lamprey (*Lethenteron camtschaticum* and *Petromyzon marinus*) *mtnr1-like* was inferred and rooted using one of the melatonin-like receptor (*mtnr-like*) genes identified in the cephalochordate amphioxus (*Branchiostoma lanceolatum*) and two of the echinoderm sea urchin *(Strongylocentrotus purpuratus)*, in order to assess the lamprey *mtnr1-like* relationships with the other vertebrate *mtnr*. A supplementary tree was inferred to analyse the relationships of the non-vertebrate *mtnr* with the vertebrate *mtnr* and amine receptors, opsin receptors and MECA receptors (25). Consensus trees were plotted using Figtree and ggtree (26).

### Synteny Analysis

Genomicus 96.01, based on data from Ensembl release 96.01, was used to examine genomic region paralogy and close gene neighborhoods (27, 28). Conserved gene families in the genomic environment of *mtnr* were investigated by manually comparing GenBank lists of gene predictions of the chromosomes carrying the *mtnr* paralogs or of conserved neighboring genes, such as fat1/2/3. Synteny analysis was performed in representatives of sarcopterygians, actinopterygians, and chondrichthyans. Representatives included: human (*Homo sapiens*), platypus (*Ornithorhynchus anatinus*), chicken (*Gallus gallus*), and western clawed frog (*Xenopus tropicalis*) for the sarcopterygians, spotted gar (*Lepisosteus oculatus*) for the actinopterygians, and Australian ghostshark (*Callorhinchus milii*) for the chondrichthyans. Gene paralogy was established for genes with common gene descriptions in the GenBank gene list and further confirmed from human or chicken orthologs using the Ohnologs browser version 1.0 (29). Families with a large number of members were excluded. We further selected representative families composed of two to four ohnologs, and having at least one member located in close vicinity of *mtnr*. The analysis was completed by Blast search in the genome assemblies to identify non-annotated neighboring genes or to confirm gene absence. Ambiguous gene identity was confirmed either by examining the surrounding genes using the Genomicus browser or by phylogenetic analysis (data not shown). Gene references are provided in Supplementary Table 2. Additional neighboring genes were investigated in teleost species including Japanese eel (*Anguilla japonica*), Asian arowana (*Scleropages formosus*), denticle herring (*Denticeps clupeoides*), zebrafish (*Danio rerio*), pike (*Esox lucius*), cod (*Gadus morhua*), and medaka. We included one pair of neighboring genes to trace the paralogy between the genomic regions derived from the 3R and genes to discriminate between the 3R *mtnr* paralog neighborhoods. The additional genes were chosen in close vicinity of *mtnr*. For the Japanese eel, genes within the genomic regions of interest were predicted using the WEBAugustus server (30). The reference and locus of genes are given in Supplementary Table 2. The location of the ancestral *mtnr* gene was found in the pre-vertebrate (pre-1R) genome and on the ancient chordate genome (31, 32). The 17 pre-1R chromosomes were reconstructed based on the identification of the ancestral tetrads, issued from the two vertebrate tetraploidizations (32). Gene families were mapped on the ancestral pre-1R genome from the pre-1R gene list available on the Genomicus web server (27, 31). The gene references are given in Supplementary Table 2. The ancestral chordate genome was reconstructed from the new version of the amphioxus genome assembly and therefore is ancestral to the pre-1R genome (32). The presumptive position of the *mtnr* ancestor was sought on the ancestral chordate genome using the Oxford plot grids of syntenies between the spotted gar, frog, chicken and human chromosomes, and the 17 chordate linkage groups (CLG), provided in Simakov et al. (32).

### Animals

Japanese medaka, *Oryzias latipes* (dr-R) were raised at 28 ± 1°C and under a photoperiod of L14:D10 at the Norwegian University of Life Sciences. Ten reproductively active adult females were individually matched with an adult male, and the 10 pairs kept in individual 1 L tanks for 1 month, then sampled at day (between 12:00 and 14:15) or at night (between 00:40 and 03:15). Medaka females were killed humanely by rapid cooling in ice water (33) after which various tissues were sampled: brain, pituitary, eye, gill, heart, spleen, adipose tissue, liver, intestine, muscle, skin, kidney, and ovary. Pituitaries were collected into TRIzol (Thermo Fisher Scientific) and stored at −80°C. The other tissues were collected in RNAlater (Ambion), incubated at 4°C overnight and then stored at −20°C. The experiment was performed in accordance with guidelines and requirements for the care and welfare of research animals of the Norwegian Animal Health Authority and of the Norwegian University of Life Sciences.

### Quantification of Gene Expression

Total tissue RNA was isolated using TRIzol followed by a DNase treatment using Turbo DNA-free kit (Thermo Fisher Scientific) (except for the pituitaries), according to the manufacturer's instructions. Complementary DNA (cDNA) was prepared from 45 ng of total RNA using SuperScript III with random hexamers (Thermo Fisher Scientific). Quantitative PCR was performed using the LightCycler96 with the FastStart Master^PLUS^ SYBR Green I (Roche). Primer sets for the medaka *mtnr1a, mtnr1b, mtnr1c*, and *mtnr1d* genes were designed using Primer3 and Vector NTI (Supplementary Table 3). Primer-set amplification efficiency was checked using serial dilutions of brain cDNA. Each sample was run in duplicate. Relative transcript abundance was calculated by comparison of the quantification cycles with efficiency correction (34). The reference genes used were *gapdh, rpl7*, and *18S* (Supplementary Figure 1). Tissue expression is presented as percentage of transcript levels per tissue for the tissue comparison and as relative transcript levels per tissue for the comparison between day and night. Expression changes between day and night were compared using the Wilcoxon rank sum test in RStudio (35). Statistical comparisons were not done for groups for which gene expression was detected in less than three out of five fish.

### PhyloFish Data Analysis

We used the PhyloFish database [http://phylofish.sigenae.org/index.html, (17)] to better understand the functional evolution of the *mtnr* genes after genome duplication in teleosts. PhyloFish is a database of *de novo* assembled transcriptome repertoires for 10 different tissues (brain, liver, gill, heart, muscle, liver, kidney, bone, intestine, gonad) across 23 different actinopterygian species. The *mtnr* were queried using TBlastN against the RNA-seq *de novo* assemblies using the PhyloFish RNA browser. RNA-seq transcripts were retrieved and the CDSs annotated for further identification by phylogenetic analysis using CLC Main Workbench. The read distribution along the transcripts was checked with the depth graphic and expression data retrieved. Relative expression of *mtnr* was calculated according to Pasquier et al. (17) as the percentage of the maximum rpkm (number of reads per kilobase per million reads).

## Results

### Vertebrate Melatonin Receptors—Gene Prediction

We screened the genomes of 70 vertebrates for melatonin receptor genes (Supplementary Table 1). Among the sarcopterygians, four genes were predicted in the coelacanth (*Latimeria chalumnae*). Among batrachian amphibians, four genes were predicted in anurans but only a single fully functional *mtnr* and two other genes with exons in opposite orientation were retrieved from an urodele, the axolotl (*Ambystoma mexicanum*) genome assembly. In Gymnophiona, the sister group of Batrachia, we identified two *mtnr* genes in the Gaboon caecilian (*Geotrypetes seraphini*). Among the sauropsids, four genes were predicted in squamates, tuatara and chelonians, but only three functional paralogs were predicted in archosaurs, along with a *MTNR* pseudogene in some, such as in the hoazin (*Opisthocomus hoazin)* and crocodile *(Crocodylus porosus*). Three genes were also predicted in mammals, including the *GPR50*. In the Tasmanian devil (*Sarcophilus harrisii*), the *MTNR1B* sequence shows an insertion in the coding sequence causing a frameshift and early stop codon, which we consider to be the result of a genome assembly error. In the platypus, the predicted *MTNR1B* shows several non-sense mutations leading to a premature stop codon. In the spotted gar, a non-teleost actinopterygian, we found three *mtnr* genes in the current genome assembly, and a fourth transcript in the PhyloFish database. In the reedfish (*Erpetoichthys calabaricus*), another non-teleost actinopterygian, four *mtnr* genes are present in the recent genome assembly. Among the basal groups of teleosts, the Elopomorpha and Osteoglossomorpha possess up to seven and five *mtnr* paralogs, respectively. We identified six paralogs in the Clupeiformes, along with two partial genes derived either from local duplication or from genome misassembly artifacts in Atlantic herring (*Clupea harengus*), and between five and seven in the Ostariophysi (excluding the polyploid cyprinidae species) (36). In the goldfish (*Carassius auratus*), up to 16 sequences coding for functional MTNR have been predicted from the genome assembly (ASM336829v1) (37). It includes several duplicates that may result from its 4R genome duplication, or allelic variants (37). In the Salmoniformes and its sister group, the Esociformes, eight (including two to three *mtnr* pseudogenes) and six paralogs, respectively, were predicted. In the Acanthomorpha, four to five functional genes were identified. Four *mtnr* genes were predicted in the two chondrichthyan orders investigated. Finally, we found a single *mtnr1-like* gene in the Agnatha. Several genes showing high sequence similarity to the MTNR were found in other non-vertebrate chordate species. In the tunicate, six genes were retrieved from the genome assembly of *Ciona intestinalis and six mtnr-like* in the cephalochordate amphioxus (*Branchiostoma lanceolatum*). We identified four genes in two ambulacrarians, in the echinoderm sea urchin (*Strongylocentrotus purpuratus*) and in the hemichordate acorn worm (*Saccoglossus kowalevskii)*. On the other hand, we could not find any *mtnr*-*like* in the genome assembly of inshore hagfish (*Eptatretus burgeri)*.

### Vertebrate Melatonin Receptors—Gene Organization

**Figure 1:**
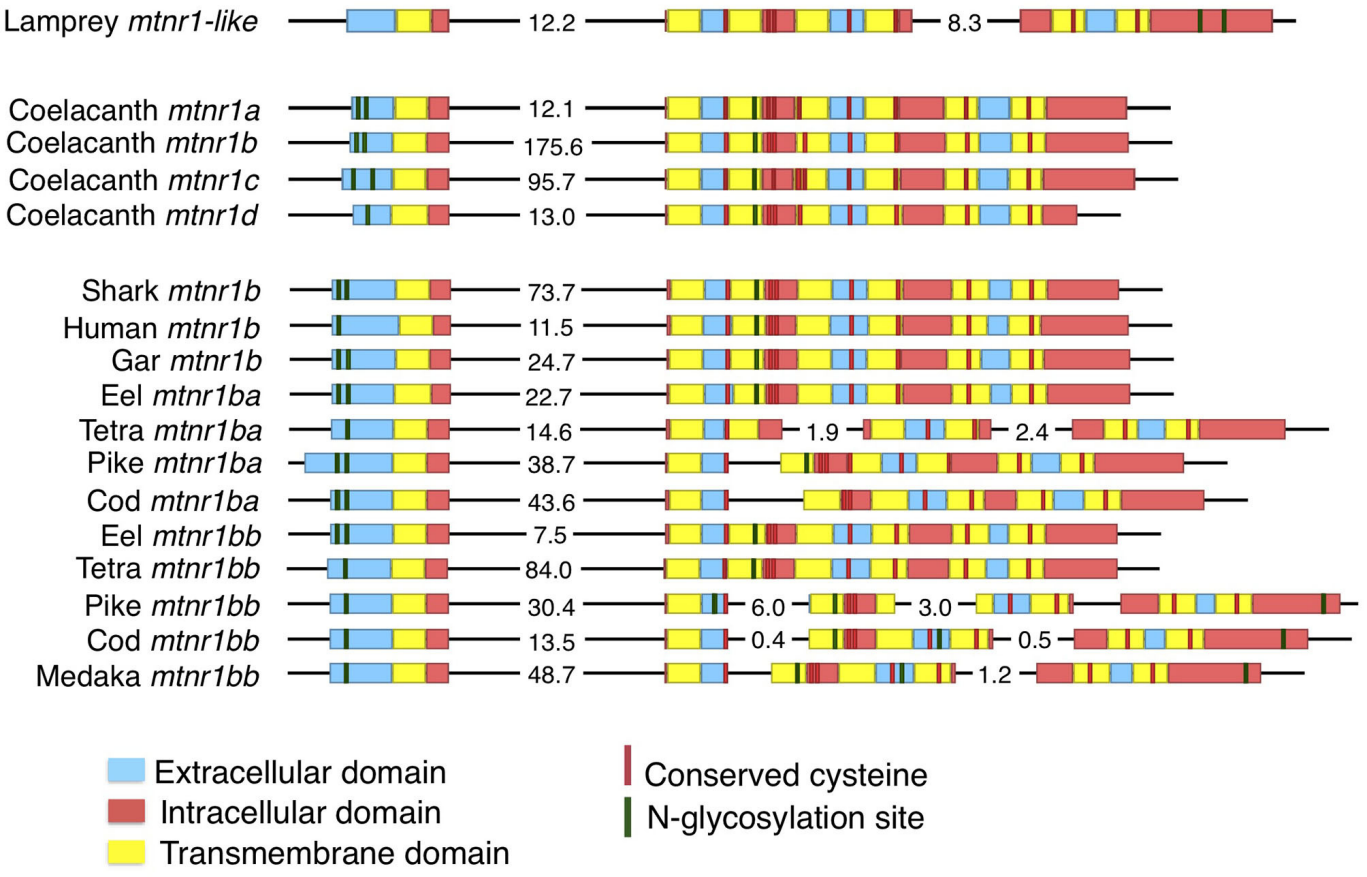
Evolution of the gene structure of *mtnr in vertebrates*. Representation of the gene structure of the lamprey *mtnr1-like*, the four gnathostome *mtnr* subtypes represented by the four coelacanth *mtnr*, and gene of *mtnr1b* duplicates in teleosts. The gene representation includes the coding sequence and its features. Genomic DNA in 5′-3′ orientation is represented by black lines, and the coding sequences with boxes. To save place, long intronic sequences have been cut and length is indicated in kb on the corresponding intron sequence. Within the coding sequences, the different domains of *mtnr1b* are represented by different colors; blue represents extracellular domains and loops, yellow for the transmembrane domain, and red for the intracellular loops and cytosolic tail. Predicted N-glycosylation sites are indicated in green, and conserved cysteine residues in red. Gene references are given in the Supplementary Table 1.

The predicted coding sequences of most of the melatonin receptors consist of two exons, interspersed by a large intron (Figure 1). The first exon codes for the N-terminal extracellular domain to the first intracellular loop, whereas the second exon encodes the six other transmembrane domains and the cytosolic tail (Figure 1). The predicted two-exon gene structure is conserved among the four MTNR subtypes in Gnathostomata. In contrast, the lamprey *mtnr1*-*like* coding sequence is organized into three exons with the first intron site common to the gnathostome *mtnr* and the second site located at the beginning of the second intracellular loop. We found intron gains in clupeocephalan *mtnr1b genes*, with one intron gained for *mtnr1ba* and up to three for *mtnr1bb*. For both pike and cod, the *mtnr1ba* additional intron shares a common insertion site, whereas the ones in the Mexican tetra (*Astyanax mexicanus*) are species-specific sites. Two of the additional introns in the Euteleostei *mtnr1bb* genes share common insertion sites with the *mtnr1ba* and the lamprey *mtnr1-like* coding sequence.

### Vertebrate Melatonin Receptors—Phylogeny

**Figure 2:**
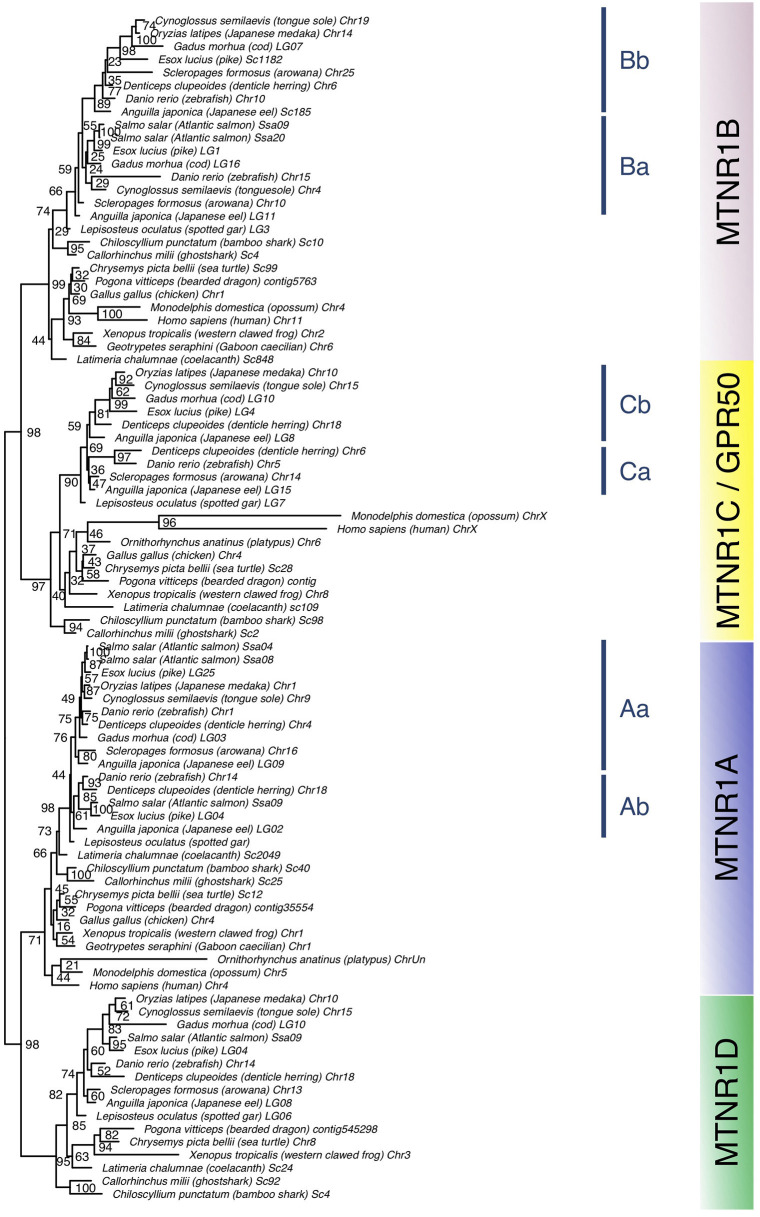
Maximum-likelihood phylogeny tree of melatonin receptors of vertebrate representatives. Melatonin receptor phylogeny was inferred from alignment of the deduced amino-acid sequences of melatonin receptor using the PhyML algorithm with the AIC selection criteria of the Smart Model Selection and the tree Subtree Pruning and Regrafting (SPR) improvement algorithm. The four gnathostome monophyletic clades are indicated with different background colors. The blue line indicates teleost melatonin receptor clades. Branch nodes are supported by bootstrap analysis with 100 replicates and only nodes with bootstrap values above 50% were considered as supported.

We obtained 290 sequences of *mtnr* from the vertebrate genomes examined (Supplementary Table 1). The phylogenetic tree divides melatonin receptors into four monophyletic groups of MTNR1A, MTNR1B, MTNR1C, and MTNR1D, well-supported for three out of the four groups (71, 99, 97, and 95% bootstrap support, respectively) (Figure 2). When including the agnathan MTNR1-like, it clusters in a position basal to the four MTNR clades (Supplementary Figure 2). Among the MTNR1A, the actinopterygian MTNR1A cluster into a single clade, with the gar MTNR1A at the basal position—in agreement with its phylogenetic position. The teleost Mtnr1a separate into two clades. For the teleost species possessing two Mtnr1a, such as Japanese eel, denticle herring, zebrafish, and pike, the duplicates are distributed into the two teleost MTNR1Aa or MTNR1Ab clades. In osteoglossomorphs and in acanthomorphs including medaka, only a single Mtnr1a was found and it clusters within the MTNR1Aa (Figure 2 and Supplementary Figure 3). The tetrapod MTNR1A cluster together (Figure 2 and Supplementary Figure 3), while the coelacanth MTNR1A branches between the gar and the chondrichthyan MTNR1A. The MTNR1B subdivide into three clades (Figure 2 and Supplementary Figure 4), including the chondrichthyan MTNR1B, which is positioned basal to the two clades of osteichthyan (bony fish) MTNR1B, in accordance with the taxonomy. Among the sarcopterygian MTNR1B, the coelacanth MTNR1B branches at the basal position of tetrapods. Among the actinopterygian MTNR1B, the gar MTNR1B is found basal to the teleost MTNR1B (Figure 2), together with the reedfish as shown in Supplementary Figure 4. The teleost Mtnr1b form one well-supported clade of MTNR1Bb (98%) and another one, gathering the other Mtnr1b, which is not well supported. In the species having two copies of Mtnr1b including eel, zebrafish, pike, cod, and tongue sole (*Cynoglossus semilaevis*), only a single copy is present in the MTNR1Bb clade. This suggests that the MTNR1B duplicates did not arise from recent segmental duplication but most probably from the teleost tetraploidization. We assumed that the second cluster of MTNR1b contains the MTNR1Ba 3R paralogs. The single medaka Mtnr1b clusters within the acanthomorph MTNR1Bb. One of the Mtnr1b paralogs in the eel and the arowana was found basal to the two clades of MTRN1B. The MTNR1C separate into two clades: one for chondrichthyans and one for osteichthyans, the latter subdividing into one clade for sarcopterygians and one clade for actinopterygians (Figure 2 and Supplementary Figure 5). The sarcopterygian MTNR1C clade, although not well-supported, includes the platypus MTNR1C and the therian GPR50 within the tetrapod MTNR1C. The latter shows long terminal branches due to the presence of elongated cytosolic tails (Figure 2 and Supplementary Figure 5). Among amphibians, we only found a single Mtnr1c in anurans (Supplementary Figure 5). Among the actinopterygian MTNR1C, the gar branches basal to the teleost MTNR1C. We found two duplicated *mtnr1c* in eels, herrings, and piranha (*Pygocentrus nattereri*) that divide into to different groups (Figure 2 and Supplementary Figure 5). The tree of *mtnr1c* including additional species did not show any clear separation between the teleost *mtnr1c* paralogs (Supplementary Figure 5). As for the MTNR1B, we defined two clades of teleost MTNR1C, the MTNR1Ca, and the MTNR1Cb, according to the eel and herring Mtnr1c/*mtnr1c* paralog distribution (Figure 2 and Supplementary Figure 5, respectively) based on the assumption that the duplicated *mtnr1c* result from the 3R tetraploidization. The single medaka Mtnr1c clusters with the other acanthomorphs into the MTNR1Cb clade. Among the MTNR1D, the chondrichthyans branch at the basal position of the osteichthyan MTNR1D (Figure 2 and Supplementary Figure 6). The coelacanth is basal to the clades of tetrapod MTNR1D, which only includes batrachian, amphibian, squamate, and chelonian MTNR1D. Among the actinopterygian MTNR1D, the gar branches basal to the teleost MTNR1D clade. The single medaka Mtnr1d branches within the other acanthomorph MTNR1D.

### Vertebrate Melatonin Receptors—Synteny Analyses

**Figure 3:**
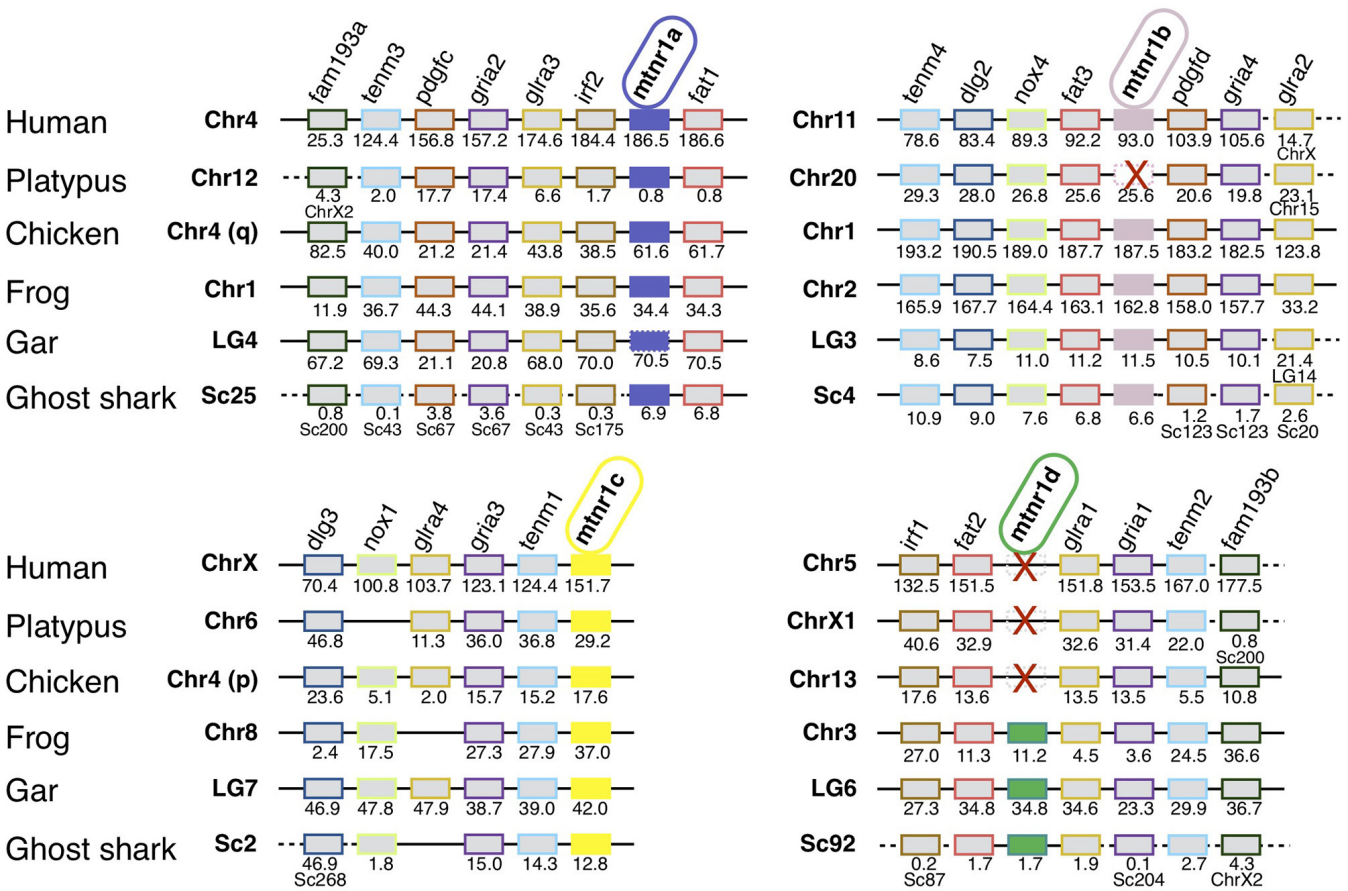
Conserved syntenic regions of melatonin receptors in gnathostomes. Locations are given in Mb. Gene order follows that of the human chromosomes 4, 5, 11, and X. Each neighboring gene family is displayed with a specific color. Red crosses indicate *mtnr* gene loss. In the spotted gar, the *mtnr1a* was found as pseudogene in the genome assembly but a transcript encoding a functional receptor was found in the PhyloFish database. We assumed that the *mtnr1a* pseudogene form results from an assembly error, and therefore *the mtnr1a* is represented by the mtnr1a blue box, but with the perimeter edges dotted. The new gene locations in the genome assembly are indicated under the gene position. Full names and references of *mtnr* and neighboring genes are given in Supplementary Table 2.

**Figure 4:**
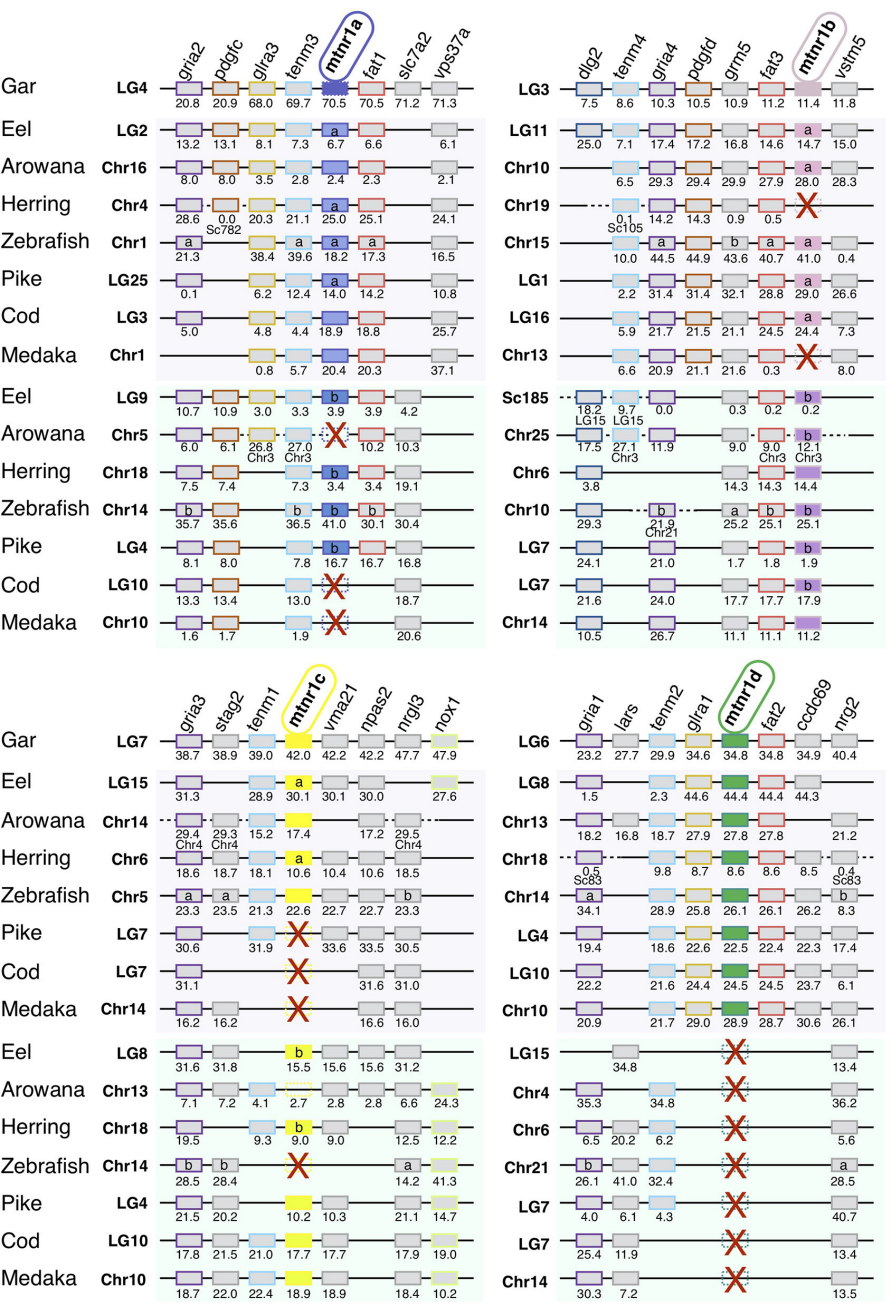
Conserved syntenic region of melatonin receptors in actinopterygians. Locations are given in Mb. Gene order follows that of the spotted gar chromosomes LG4, LG3, LG7, and LG6. Each neighboring gene family is displayed with a specific color. Red crosses indicate *mtnr* gene loss. In the spotted gar, the *mtnr1a* was found as pseudogene, in the genome assembly but a transcript encoding a functional receptor was found in the PhyloFish database. We assumed that the *mtnr1a* pseudogene form results from an assembly error, and therefore the *mtnr1a* is represented by the mtnr1a blue box, but with the edges dotted. The new gene locations in the genome assembly are indicated under the gene position. Full names and references of *mtnr* and neighboring genes are given in Supplementary Table 2.

We identified 63 conserved gene families on the chromosomes carrying a *mtnr* gene, shared by the gar and human genomes (Supplementary Figure 7). Figure 3 shows the representative neighboring gene families of *mtnr* that have members in close vicinity of a *mtnr:* dlg2/3, f*am193a/b, fat1/2/3, glra1/3/4, gria1/2/3/4, irf1/2, pdgfc/d*, and *tenm1/2/3/4* paralogs. The localization of the members of these gene families reveals that *mtnr1a, mtnr1b, mtnr1c*, and *mtnr1d* are maintained in conserved ohnologous chromosomal regions (paralogons) in human, chicken, frog, gar, and shark (Figure 3), supporting the *mtnr* partition into 4 clades in the gnathostomes. In human, platypus and chicken, the gene environment of *mtnr1d* is well-conserved—but the *mtnr1d* itself is absent. The comparisons of gene family member distribution indicate a high retention of ohnolog genes on the chromosome carrying the *mtnr1a* in both gar and human genome (80% for LG4 and 85% for Chr4, respectively) (Supplementary Figure 7). The gene retention falls to 68% for the chromosome carrying the *mtnr1d* gene environment (LG6 and Chr5), to 33–48% and 25–32% for the chromosomes carrying the *mtnr1b* (LG3 and Chr11) and the *mtnr1c* (LG7 and ChrX) in the gar and human genome, respectively. Gene synteny comparison between actinopterygians (Figure 4) reveals that the four *mtnr* paralogons were duplicated in teleosts, in agreement with the 3R teleost tetraploidization. The neighboring genes of *mtnr1a, mtnr1b, mtnr1c*, and *mtnr1d* in gar are conserved in the 3R-duplicated genomic regions. In the species having the *mtnr* in duplicates, each copy was located on one 3R-duplicated genomic region. Whereas, the four *mtnr* paralogs are localized on four different chromosomes in gar, we found the paralogons of *mtnr1c* and *mtnr1d* on the same duplicated linkage group in the teleost species examined (with the exception of zebrafish). In the clupeocephalans, including the herring, zebrafish, pike, cod, and medaka, *mtnr1ab* and *mtnr1d* lie on the same chromosome (Figure 4 and Supplementary Figure 7). In addition, the genomic region of *mtnr1bb* is fused to the genomic region of *mtnr1ca* in herring, pike, cod and medaka.

**Figure 5:**
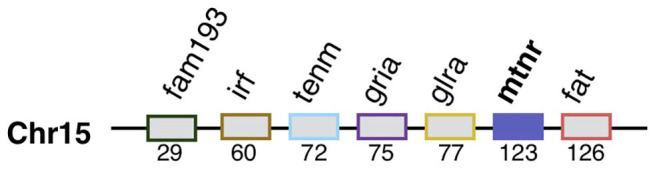
Mtnr gene neighborhood in the pre-vertebrate genome. *Mtnr* and its syntenic genes were mapped from the gene list of the pre-1R ancestral genome reconstruction (Genomicus 69.10). The gene pre-1R gene list is available for download on the Genomics webserver and (115). The gene order is relative.

The screening of the pre-1R genome (31) reveals that the *mtnr* as well as most of the gene families syntenic with *mtnr* derived from the duplication of ancestral block localized on the pre-1R chromosome 15 (Figure 5 and Supplementary Table 3). Comparison of the localization of orthologous genes between the *mtnr-*bearing chromosomes in human, chicken, frog and gar, and the 17 CLG suggests the chordate linkage group F (CLGF) to be the ancestral chromosome where the ancestral *mtnr* was located.

### Vertebrate Melatonin Receptors—Day/Night Tissue Distribution of Gene Expression

**Figure 6:**
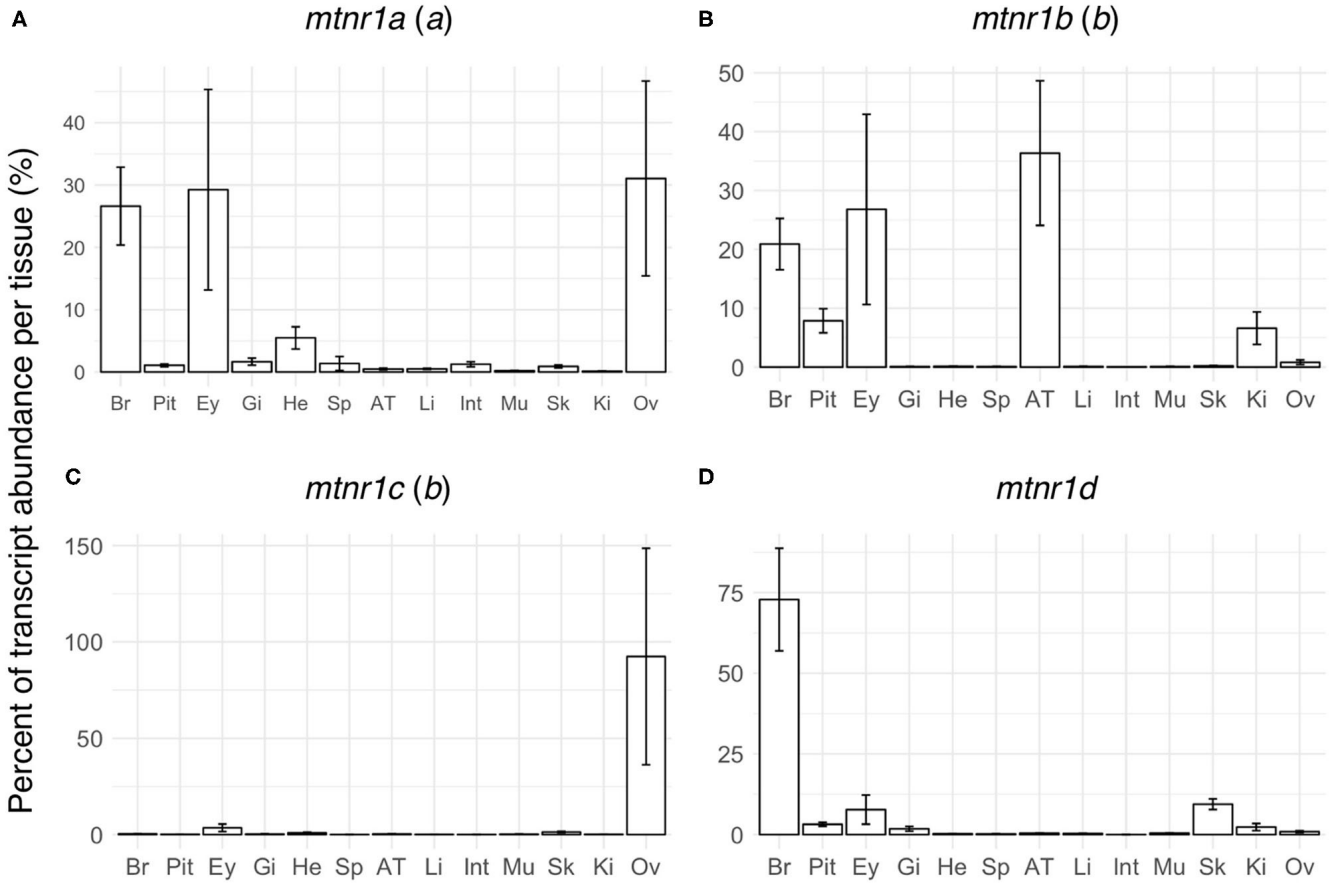
Tissue distributions of the four melatonin receptors in reproductively active medaka females. Gene expression profiles of the four melatonin receptor paralogs: **(A)** *mtnr1a*, **(B)** *mtnr1b*, **(C)** *mtnr1c*, and **(D)** *mtnr1d* in brain (Br), pituitary (Pit), eye (Ey), gill (Gi), heart (He), spleen (Sp), adipose tissue (AT), liver (Li), intestine (Int), muscle (Mu), skin (Sk), kidney (Ki), and ovary (Ov) of reproductively active medaka females. The bar plots represent the relative expression calculated as the percentage of the mean of transcript levels (mean ± se) per tissue. The letter inside the parentheses indicates the 3R paralog identity.

**Figure 7:**
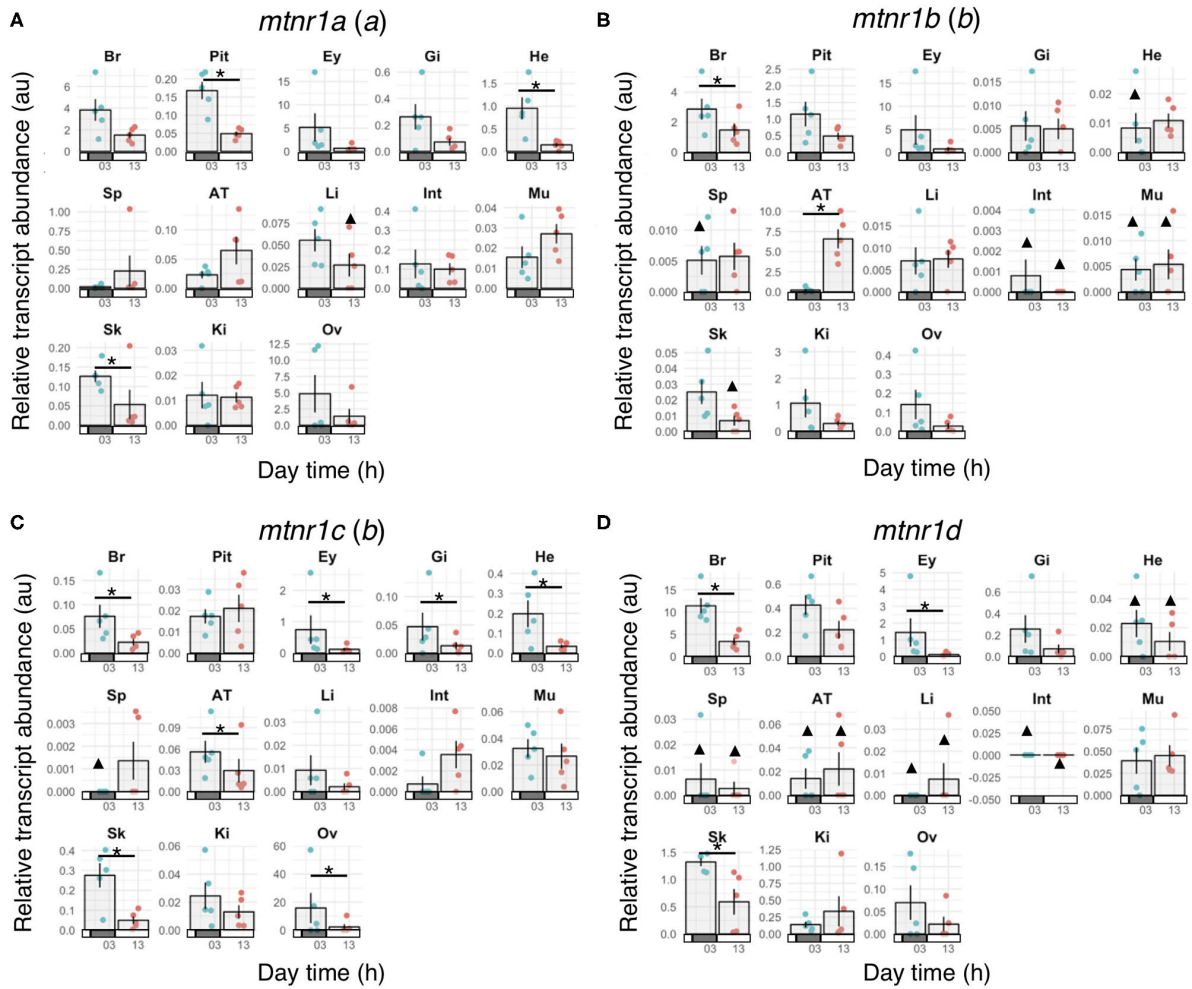
Melatonin receptor expression profiles in mature female medaka during the day and night. Gene expression of the four melatonin receptor paralogs: **(A)** *mtnr1a*, **(B)** *mtnr1b*, **(C)** *mtnr1c*, and **(D)** *mtnr1d* in brain (Br), pituitary (Pit), eye (Ey), gill (Gi), heart (He), spleen (Sp), adipose tissue (AT), liver (Li), intestine (Int), muscle (Mu), skin (Sk), kidney (Ki), and ovary (Ov) of reproductively active medaka females. Fish were maintained under a prolonged photoperiod cycle of L14:D10 (light from 08:00 to 22:00) for one month prior to sampling and sampled either during the day (around 13:00) or the night (around 01:00) (*n* = 5 per group). Bar plots represent the mean ± se of the relative transcript levels for the 5 fish and the dots show the individual data points depending on the sampling time. Night sampling is indicated with blue dots and day sampling with pink dots. Bars along the x-axes represent the light phase (open bars) and dark phase (solid bars). Statistically significant changes in gene expression between day and night (Wilcoxon rank sum test) are indicated by an asterisk (*). Triangles (▴) indicate groups for which gene expression was detected in less than 3 out of 5 fish. Statistical comparisons were not done for these groups. The letter inside the parentheses indicates the 3R paralog identity.

In order to establish whether *mtnr* paralogs diverged functionally, we studied their expression patterns in the medaka. The four receptor transcripts showed differential tissue distribution in adult female medaka (Figure 6). All *mtnr* genes were found to be expressed, but at different levels in the brain and in the eye. *Mtnr1a* is widely expressed, being detected at high levels in the brain, eye, ovary and heart, and at lower levels in the other studied tissues. We found high levels of *mtnr1b mRNA* in the brain, pituitary, eye, adipose tissue, and kidney. The highest expression of *mtnr1c* was measured in the ovary, but it is also expressed at lower levels in the brain, eye, gill, heart, adipose tissue, muscle, and kidney. The main sites of *mtnr1d* expression are the brain, pituitary, eye, and skin. Comparison of the transcription levels during the day and the night in female medaka mainly revealed that expression was up-regulated during the night, in a paralog-specific and tissue-specific manner (Figure 7). We found significantly higher expression levels in the brain for *mtnr1d* (3.3-fold), in the pituitary for *mtnr1a* (2.5-fold), in the eye for both *mtnr1c* and *mtnr1d* (6.3- and 11.9-fold, respectively), in the heart for *mtnr1a* (6.5-fold), and in the skin for both *mtnr1c* and *mtnr1d* (5.5 and 2.2-fold, respectively). In contrast, transcript levels were significantly higher during the day in adipose tissue for *mtnr1b* (30.8-fold).

**Figure 8:**
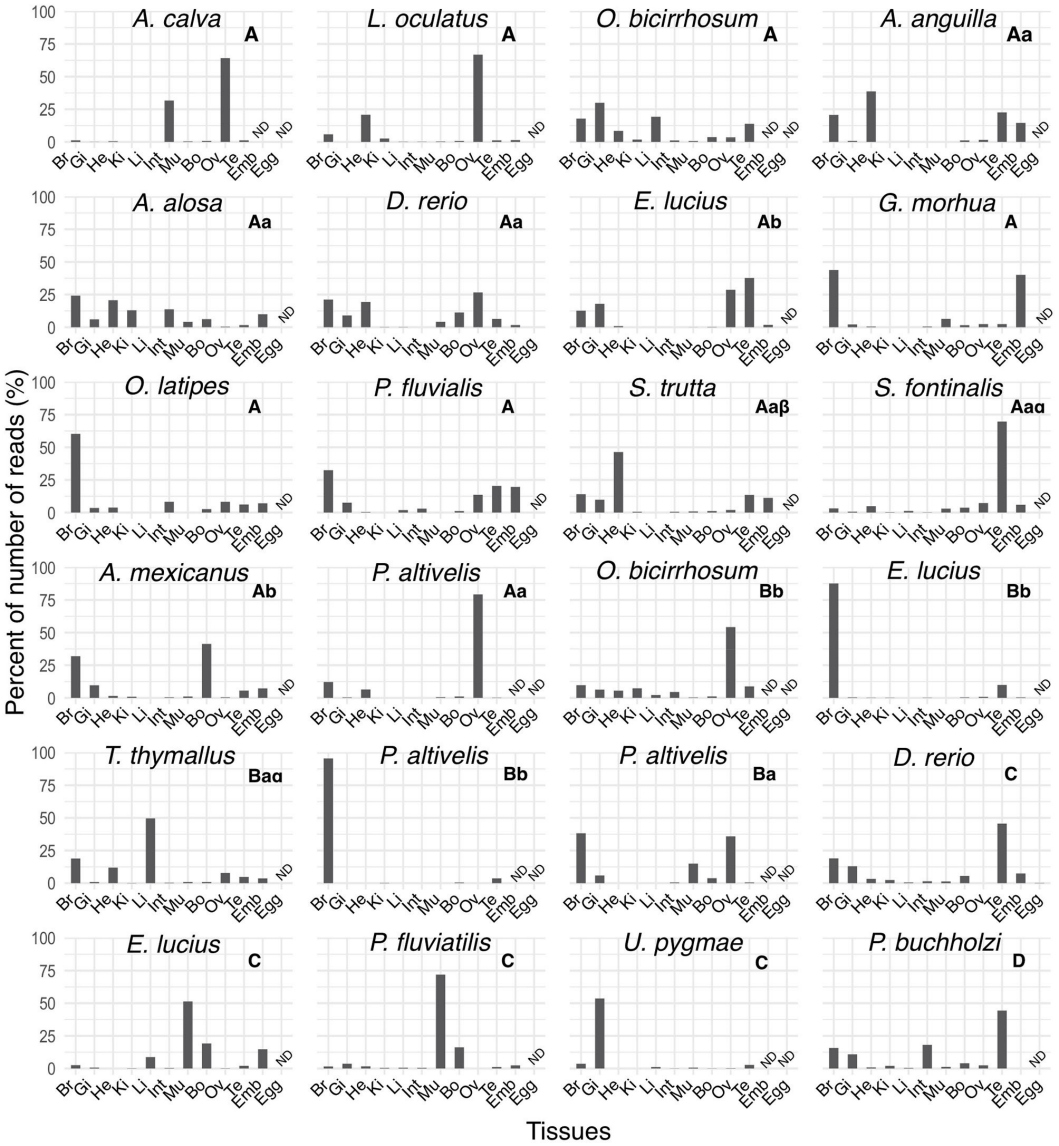
Tissue distribution profiles of melatonin receptors in different ray-finned fish in the PhyloFish database. The bar plots represent the relative expression calculated as the percentage of the maximum rpkm (number of reads per kilo base per million reads) value per species. The *mtnr* transcripts were found by exploring the PhyloFish database (17) and transcript identity was established by phylogenetic analysis (data not shown). Subtype identity is given at the top of each graph. ND indicates a tissue for which data were not available.

We compared the expression distribution of *mtnr* paralogs across actinopterygian species using the PhyloFish database (Figure 8). We identified 23 *mtnr* transcript sequences including 14 corresponding to *mtnr1a*, five for *mtnr1b*, four for *mtnr1c*, and a single *mtnr1d*. Different expression profiles were observed between *3R mtnr orthologs*. The major sites of *mtnr* expression are the brain and the gonads, but a few *mtnr* were also found in other peripheral tissues such as heart, gill, and muscle. Among the four subtypes, only *mtnr1a* was found to be expressed in the two non-teleost actinopterygians, the gar and the bowfin (*Amia calva*), with high expression in ovary (Figure 8). A single pair of conserved 3R-paralogs was identified for the *mtnr1b* in ayu (*Plecoglossus altivelis)* and showed differential expression patterns, with *mtnr1ba* found in the brain and several peripheral tissues, and *mtnr1bb* almost exclusively expressed in the brain.

## Discussion

### Structure of Melatonin Receptor Genes

In this study, we identified the *mtnr* repertoire of 70 representatives of all living gnathostome lineages. Comparison of *mtnr* gene organization revealed a main shared gene structure, with two exons separated by a large intron for the four gnathostome receptor paralogs. The lamprey *mtnr1*-*like* gene is organized into 3 exons. One of its intron sites is shared with the gnathostome *mtnr*, suggesting that this intron was inherited from the ancestral vertebrate *mtnr*. The two-exon gene structure was conserved in the *mtnr* 3R-paralogs in teleosts, except for the *mtnr1b* 3R-paralogs in the clupeocephalans, which show several intron insertions. Introns are known to affect transcription and translational processing efficiency of numerous genes (38). It is assumed that intron gain and loss plays an important role in the divergence of duplicated genes (39). Several other GPCR gene families show novel intron insertions in the transmembrane domain in Euteleostei (40–42). Comparison of the insertion sites shows that sites were conserved between the independent intron gain events, suggesting either a conserved mechanism or functional constraints at the origin of intron gains in the *mtnr1b*. One major mechanism could be related to the combination of conservation of donor/acceptor sites and the increased transposon activity that activates double-strand break repair (43, 44).

### Vertebrate Tetraploidizations at the Origin of the Melatonin Receptor Family

Our phylogenic analysis divides the melatonin receptors into four gnasthostome paralogs, which is in agreement with the recent classification of the melatonin receptors into MTNR1A, MTNR1B, MTNR1C, and MTNR1D (also named MTNR1A-like subtypes and including the teleost *mtnr1a2* and *mtnr1a1.4*) (10–12). Several branching incongruities were observed, such as the position of coelacanth MTNR1A, which may result from the adverse effect associated with the heterogenous evolution rate of protein-coding genes among gnathostomes (45–48).

**Figure 9:**
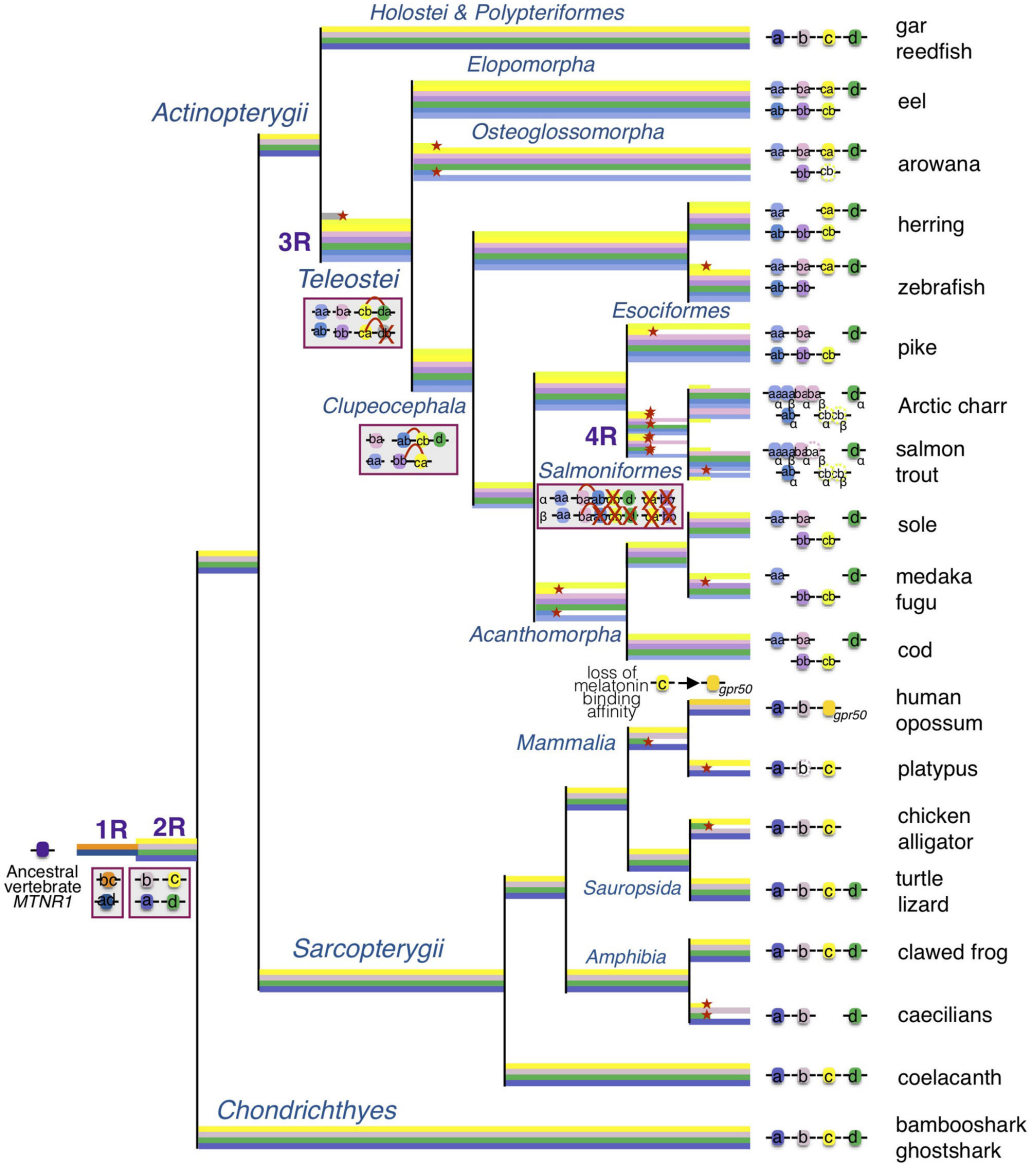
Evolutionary scenario of the melatonin receptors in vertebrates. This evolutionary scenario was developed on the basis of the phylogeny and synteny analyses (Figures 2–4 and Supplementary Figures 2–5). The four receptor genes are derived from duplication of an ancient *mtnr1* gene through vertebrate tetraploidization (1R and 2R). The teleost 3R event generated duplicates of the four *mtnr* subtypes. Multiple and selective gene losses occurred, leading to *mtnr* gene repertoires differing between the main gnathostome lineages. Genome tetraploidization events (1R, 2R, 3R, and 4R) are indicated in purple. Major gene gain and loss events as well as chromosome rearrangements are indicated in red boxes. Genes located on the same linkage group are represented by clusters on the genomic DNA line. Red crosses indicate gene loss. The red arc indicates genomic region fusion events. Colored tree branches represent *mtnr* gene lineages. Red star * on a tree branch indicates gene loss. The receptor identities are indicated in or beside the boxes. Paralogs originating from teleost 3R are designated by a and b suffixes, and salmonid 4R paralogs are designated by α and β suffixes.

The identification in lamprey of a single *mtnr1*-*like* gene, surrounded by genes similar to a combination of genes of the gnasthostome MTNR genomic environment, supports a common ancestral origin of the vertebrate *mtnr* gene family (12). However, the comparison of the local gene neighborhood revealed that the *mtnr* genes evolved in a different gene environment in gnathostomes. Only the *fat* gene family was found conserved, with a *fat* gene positioned in tandem with 3 of the 4 *mtnr* paralogs (*mtnr1a, mtnr1b*, and *mtnr1d*). The small scale synteny analysis did not provide sufficient information to demonstrate the paralogy of the *mtnr* genomic regions and subsequently to draw any inference on the evolutionary events that gave rise to the *mtnr* family. To better understand the relationships between the four *mtnr* genes, we investigated the conserved gene families having a member in the *mtnr* neighborhood at the chromosome scale in gar and human genome. From this analysis, we chose 10 gene families of four to two ohonologous genes showing members in the vicinity of at least one *mtnr* in different vertebrate representatives. Several of these gene families have already been studied and were supposed to be derived from the vertebrate tetraploidizations: TEMN (49, 50), GLRIA (51), FAT (12, 52), NOX (53), DLG (54), and IRF (55). The genome mapping of the members of these gene families reveals that the four *mtnr* paralogs reside on four independent ohnologous chromosomal regions. In chicken, both *mtnr1a* and *mtnr1c* are located on chromosome 4 which results from the fusion of the ancestral avian chromosomes 4 (4q) and 10 (4p) (56). Based on our observations, we propose as the most parsimonious evolutionary scenario, that the genomic regions containing the *mtnr* originated from the two rounds of vertebrate tetraploidization (1R and 2R) and subsequently from the teleost tetraploidization (3R). Therefore, we infer that the *mtnr* gene family derived from the duplication of an ancestral *mtnr* gene during the two vertebrate tetraploidizations (1R and 2R, Figure 9).

We attempted to establish the location of the ancestral *mtnr* gene on the pre-vertebrate and the ancestral chordate genomes (31, 32). The ancient chordate genome was reconstructed based on the new amphioxus genome (32), and is ancestral to all the chordates. The pre-1R genome was inferred from the tetrads of contiguous ancestral regions identified in the ancestral amniote genome, and is ancestral to those chordates that experienced 1R (and subsequent) genome duplication, leading to the vertebrates. It excludes the amphioxus and tunicate chordates. The ancient chordate genome is therefore ancestral to the pre-1R genome. The search in the pre-1R genome reconstruction revealed that the four *mtnr* genes descend from an ancestral *mtnr* gene localized with the ancestors of several extant syntenic genes on the pre-1R chromosome 15. The ancient genomic region including the ancestor of *mtnr* may reside on linkage group F of the ancestral chordate genome reconstruction (32). Analysis of the conserved syntenies between the CLG ancestry and vertebrate chromosomes suggests that the first ancient vertebrate duplication (1R) occurred by autotretraploidization producing chromosome pairs 1-2 and the second (2R) by allotetraploidization leading to asymmetrical pairs α-β showing a higher gene retention on the α segment than on the paralogous β. According to this scenario, the 1R duplication of an ancient *mtnr* on CLGF gave rise to the ancestor of *mtnr1b/c* and *mtnr1a/d* (Figure 9). This is in agreement with the MTNR relationships established in our phylogeny and synteny analyses. The auto-allo-tetraploidzation scenario suggests that the *mtnr1a* and *mtnr1b* are localized on α segments, and the *mtnr1d* and *mtnr1c on* β segments (32). The analysis of *mtnr* ohnolog gene family conservation shows that the chromosome carrying the *mtnr1a* has maintained a higher number of ohnolog genes suggesting that its genomic environment preserved a more ancestral state. All these observations corroborate our evolutionary scenario for the vertebrate *mtnr*.

### A Single Melatonin Receptor in Cyclostomata

The timing of the divergence between Gnathostomata and the Cyclostomata is still under debate. Comparison of the lamprey genome with the ancestral amniote genome reconstruction places the divergence of lampreys and jawed vertebrates after the second vertebrate tetraploidization event (2R), about 450 million years ago (31). The chromosomal synteny comparison between amphioxus and the vertebrate genomes indicates that the cyclostomes experienced the first vertebrate tetraploidization (1R) about 490 million years ago, before they split from the Gnathostomata (32). In the first scenario, the ancestor of the extant cyclostomes might have possessed the four vertebrate *mtnr*. Only a single *mtnr* was found in the genome of lampreys suggesting the loss of three *mtnr* paralogs. In the second scenario, the lamprey might have possessed two vertebrate *mtnr* and may have lost one of them. The evolutionary tree did not allow us to resolve the identity of the lamprey *mtnr1*-*like*. This suggests that the primary structure of the lamprey MTNR1-like might both conserve ancestral features but have diverged during the lamprey radiation. No *mtnr1*-*like* gene could be found even in the vicinity of any *fat* gene locus in the genome assembly of another agnathan, the inshore hagfish. The identification of a single *mtnr1*-*like* gene in lamprey and the absence of *mtnr1*-*like* gene in hagfish is in agreement with a previous *in vitro* autoradiography study using 2-iodomelatonin that revealed the presence of melatonin binding sites in the brain in lampreys but was unsuccessful in the Atlantic hagfish (*Myxine glutinosa*) and in amphioxus (57). On the other hand, we identified several sequences related to the *mtnr* in amphioxus that cluster with the tunicate, acorn worm, and sea urchin *mtnr-like* sequences in a sister clade of non-vertebrate *mtnr* (Supplementary Figure 8). This is in agreement with the reports of *mtnr*-*like* genes in urochordate, cephalochordate, and hemichordate genomes (58–60). The melatonin receptors are assumed to have arisen from the duplication of a common ancestor between melatonin receptor and opsin genes in a eumetazoan (61). Melatonin has been identified in diverse non-vertebrate groups, including Echinodermata (62), Arthropoda (63–65), and Cnidaria (66). A study using [125I]-melatonin binding to eyestalk membrane revealed the presence of melatonin high affinity binding sites in the retinular photoreceptors in crayfish (*Procambarus clarkii*) (67). The presence of GPCR with pharmacological properties similar to the vertebrate MTNR has been observed in non-vertebrates: MTNR1A (MT1) in *Caenorhabditis elegans* (68) and MTNR1B (MT2) in crayfish (67). In addition, melatonin signaling via a MTNR-like has been shown to play a key role in the circadian control of ciliary swimming in the marine annelid, *Platynereis dumerilii* through the stimulation of cholinergic ciliomotor neuron activity (69).

The role of melatonin as the modulator of circadian and seasonal rhythmicity is assumed to have emerged in vertebrates with the functional shift of the AANAT from a role in detoxification to a role in melatonin synthesis and the evolution of the pineal gland (70). Both hagfish and amphioxus lack a pineal gland (71). The amphioxus possesses only a non-vertebrate AANAT type (70), and to our knowledge no AANAT has been found yet in hagfish. This opens up the question about the role, the regulation, and the biosynthetic pathways of melatonin as well as its signaling pathways in non-vertebrate bilaterians. To date, no studies have examined the capacities of these non-vertebrate MTNR-like receptors to respond to melatonin. Further investigations are needed to determine whether the MTNR-like receptors are functional and are involved in melatonin signaling in non-vertebrate species.

### Conservation of Melatonin Receptor Repertoire in Chondrichthyes

Little is known about the role of melatonin in chondrichthyans, however their genomes do encode all of the four *mtnr* paralogs. Studies indicate that photoperiod may act as potential driver in the control of seasonal and diurnal movements in sharks and rays [for review see (72)]. An *in vitro* autoradiography study using 2-iodomelatonin has revealed the presence of melatonin binding sites in the brain in skates (57), and recent studies have shown that melatonin stimulates luminescence of a ventral photogenic skin area in three lantern sharks (Etmopterus) and pygmy shark (*Squaliolus aliae*) (73–76). Exploration of the lantern shark transcriptome revealed the expression of *mtnr1c* paralog (GHAY01039059) in the ventral skin (77).

### Evolution of Melatonin Receptor Repertoire in Sarcopterygii

Four melatonin receptor subtypes were conserved in the coelacanth. Among amphibians only the anurans conserved the four vertebrate MTNR subtypes. In the batrachian urodele, the axolotl, we found three *mtnr* genes but only *mtnr1d* was found to encode a functional receptor. Although both *mtnr1a* and *mtnr1b* are present in the axolotl genome assembly, they exhibit opposite exon directions, preventing transcription of these two genes. We found only the second exon of *mtnr1b* predicted as *mtnr* in the Ambystoma mexicanum assembly in UCSC. Tissue-specific gene expression data for axolotl (available on the UCSC Genome Browser) revealed high transcription levels of *mtnr1b* gene in the ovary suggesting that the axolotl genome does encode a functional Mtnr1b receptor. The exon inversion observed for both *mtnr1a* and *mtnr1b* genes might be an assembly artifact caused by the presence of large introns and high number of a large repetitive sequences in the very large axolotl genome (78, 79). We did identify the four *mtnr* chromosomal regions in the Gabon caecilian (*Geotrypetes seraphini*) genome assembly, but both *mtnr1c* and *mtnr1d* genes are lacking. As in other vertebrates, melatonin regulates pleiotropic actions in amphibians. It has been shown that photoperiod has an effect on the growth and the development of tadpoles (80), and administration of melatonin accelerates metamorphosis (81). Melatonin has been shown to be involved in the direct regulation of body blanching in axolotl and anuran tadpoles, a background adaptation response, resulting from the contraction of dermal melanophores in response to a dark environment. The *mtnr1c* (named *mel1c*) was originally cloned from skin melanophores of xenopus (*Xenopus laevis*), suggesting it mediates the melatonin effect on pigment aggregation in these cells (3). The loss of *mtnr1*c, and potentially *mtnr1a* in axolotl raises the question how it has impacted the response to daily and seasonal rhythms in the evolutionary history of urodeles. Most of the caecilians (Gymnophiona) are subterranean, fossorial, and limbless amphibians living in tropical soils. They are little exposed to light and only show rudimentary eyes that evolved rod-opsin-only retina (82). However, little is known about the mechanisms and signaling related to the light perception in this group. Our results suggest there might be a relationship between the adaptation to subterranean habitat and the loss of melatonin signaling mediated by the Mtnr1c and Mtnr1d in caecilian species.

Among the sauropsids all the four *mtnr* genes were present in lizards and turtles. However, as *Mtnr1d* was only found as a pseudogene near the *Fat2* locus in a few birds and a crocodilian species, it seems that the loss of *Mtnr1d* predates the emergence of birds, and might have occurred in the ancestor of the archosaur lineage (Figure 9). Our research in mammals revealed that two functional melatonin receptors, the MTNR1A and the MTNR1B, as well as the orphan GPR50, are conserved in most therians. We identified two functional MTNR in the platypus genome; in our phylogenetic tree, platypus MTNR1C was positioned as being orthologous to the therian GPR50, in agreement with previous studies (6, 12, 25, 83). The second platypus paralog clusters with the MTNR1A in the gnathostome MTNR tree (Figure 2), however when the lamprey MTNR1-like are included, it branches at the basal position of MTNR with the lamprey MTNR1-like (Supplementary Figure 2). Genomic location analysis reveals that the latter is surrounded by genes syntenic with *MTNR1A*, confirming its identity as MTNR1A members (12). Sequence comparison indicates that the predicted platypus MTNR1A is strongly derived, which may have led to misplacement due to long branch attraction. The platypus *MTNR1B* shows several non-sense mutations in the coding sequence suggesting the *MTNR1B* is experiencing pseudogenization. Two non-sense mutations were also found in the seasonally breeding Siberian hamster *MTNR1B* (named Mel1b), implying that the control of seasonal breeding might be exerted through MTNR1A activation (84). In several species of Cetacea, both MTNR1A and MTNR1B were lost or inactivated (85). The disruption of melatonin signaling is associated with the acquisition of an unihemispheric sleeping lifestyle and the development of long-term vigilance (85). The apparent absence of *mtnr1d* in all mammalian genomes examined, even in metatherians and in monotremes, confirms the loss of *mtnr1d* in the mammalian lineage (11, 12). So far, *mtnr1d* had only been found missing in birds and mammals (11, 12). The function of MTNR1D was therefore inferred among convergent traits specifically shared by birds and mammals (e.g., acquisition of homeothermy) that may have driven to the reorganization of the melatonin response pathway, contributing to the loss of the *mtnr1d* gene (12). The discovery of *mtnr1d* loss in other taxa, including crocodilians and caecilians, reopens the question about the role of MTNR1D in gnathostomes.

### Expansion of Melatonin Receptor Repertoire in Actinopterygii

Our phylogeny and synteny analyses, which included several basal new teleost species, provide a more complete evolutionary scenario of *mtnr genes* in actinopterygians than the ones recently proposed (11, 12). We identified the four functional MTNR subtypes in two non-teleost actinopterygians, the reedfish and the spotted gar. For the latter, this includes *mtnr1a* retrieved from the PhyloFish database that encodes a fully functional receptor. Previously found as a pseudogene trace in the spotted gar genome, *mtnr1a* was assumed to have become non-functional in the holostei lineage (12). Our results show that the four functional vertebrate *mtnr* genes have been conserved in two sister taxa of non-teleost actinopterygians, the Polypteryformes, and the Holostei. Most teleosts have conserved each MTNR subtype, either as a singleton or in duplicate. The reduction in number of *mtnr* 3R-paralogs could have occurred according to different scenarios of conservation. The synteny conservation between gar and human genomes suggests that the teleost genome duplication accelerated gene loss and genome reshaping (45, 86). Only *mtnr1d* was maintained as a singleton in teleosts, suggesting early loss of one of the 3R *mtnr1d* paralogs after 3R and before the teleost radiation (Figure 9). Likewise, the presence of only one copy of *mtnr1d* in the salmonid lineage indicates the early loss of one of the 4R-*mtnr1d* paralogs after the salmonid tetraploidisation 4R (10, 12). All the teleost species have conserved the *mtnr1aa* paralog whereas the *mtnr1ab* was lost twice independently, in the osteoglossomorphs and in the acanthomorphs. In our phylogenetic trees (Figure 2 and Supplementary Figure 4), the resolution of the branching of teleost pairs encoding Mtnr1b was low for one paralog, preventing its identification. However, the synteny analysis demonstrates that most teleost species retained the 3R duplicated pair of *mtnr1b*, and allows the accurate assignment of the *mtnr1b* paralog identity. The loss of one 3R paralog seems to have occurred more recently as the *mtnr1ba* was lost in the clupeiforms and in several percomorph lineages, such as the Ovalentaria (medaka) and the *mtnr1bb* in the salmonids. Previous analyses of MTNR1C phylogeny and synteny suggest that the presence of a single copy of *mtnr1c* resulted after the loss of one paralog early after the 3R event as for *mtnr1d* (12). In contrast, here we show that both 3R paralog copies are still present in a few teleost lineages, including eels, herrings and piranha. The synteny analysis using basal teleost representatives reveals that the single copy of *mtnr1c* resulted from the lineage-specific loss of either one of the 3R paralogs: the zebrafish and arowana lost the *mtnr1cb* and the acanthomorph lost the *mtnr1ca*.

The salmonids have conserved only three MTNR subtypes, including duplicates of *mtnr1aa* and *mtnr1ba* genes in Arctic char (*Salvelinius alpinus*) and coho salmon (*Oncorhynchus kisutch*), that derived from the salmonid tetraploidization 4R (Figure 9) (10). In Atlantic salmon and rainbow trout (*Oncorhynchus mykiss*), the 4R paralog of *mtnr1ba*β was considered to be a pseudogene, as the two first exons of *mtnr1ba*β were found 6 Mbp apart in Atlantic salmon (10) and it contains an inversion of the first exon preventing transcription in rainbow trout. Transcriptomic data mining returned two sequences for the *mtnr1ba*α 4R paralog (GBRB01031846 and GGAQ01003590) and no sequence for the *mtnr1ba*β in Atlantic salmon. The inactivation of *mtnr1ba*β 4R paralog seems to be species-specific. Changes in the melatonin pathway may have been the source of functional redundancy of 4R *mtnr1ba* paralogs and would have favored the loss of *mtnr1ba*β in trout and salmon. The salmonids have retained *mtnr1c* only in the pseudogene form. Surprisingly, we identified a *mtnr1c* sequence (GBRB01047133) showing high identity with *mtnr1c* (LOC106603297) in the Atlantic salmon transcriptome database. Further investigations are needed to determine whether the salmon degenerate *mtnr1c* gene plays a role in the regulation of melatonin response. Together, our results demonstrate that the diversity of the teleost mtnr repertoire has been shaped by lineage-specific gene losses during the teleost radiation.

Our synteny analysis revealed chromosome rearrangements between the *mtnr1a, mtnr1c*, and *mtnr1d* paralogons. Whereas, the four receptor paralogs reside on different chromosomes in gar, *mtnr1c* and *mtnr1d* co-localize on two 3R-duplicated genomic regions in teleosts, suggesting that a genomic rearrangement, merging the *mtnr1c* and *mtnr1d* paralogons, took place after the teleosts split from the holosteans, and before 3R (Figure 9 and Supplementary Figure 9). This is in agreement with the assumption of the occurrence of intensive interchromosomal fusions prior to the teleost tetraploidization (87, 88). In other hand, as only *mtnr1ab* shared chromosome with *mtnr1d*/*mtnr1cb*, the fusion between the two paralogons might be subsequent to 3R and prior to the clupeocephalan radiation. The co-localization of *mtnr1bb* and *mtnrca* genomic regions in clupeiform and euteleost species suggests another chromosomal fusion between the region containing the *mtnr* genes prior to the clupeocephalan radiation (Supplementary Figure 9). These observations suggest that further chromosomal fission and fusion events occurred in the clupeocephalan ancestor leading to additional chromosomal co-localizations of *mtnr* paralogs. This is in agreement with the comparison of interchromosomal rearrangement rates between chicken, gar and several teleost species including zebrafish, stickleback, pufferfish and medaka, which reveals higher rates of fission/translocation after 3R in some lineages (16, 88). The loss of 3R paralogs and the genomic rearrangements observed for the *mtnr* seem to have occurred independently from each other.

### Day/Night Tissue Expression of Melatonin Receptors in Female Medaka

Medaka only retained one of the 3R paralogs of each vertebrate *mtnr* gene. The four receptors are able to respond specifically to melatonin by eliciting the inhibition of cAMP via inhibitory G protein in transfected Hepa-E1 cells (11). Studies have shown that seasonal and daily photoperiod changes affect physiological functions and behavior in medaka. Shortened photoperiod alters female fecundity and male behavior (89), while treatment with melatonin induced gonadal regression and lipid biosynthesis suppression (90). In addition, light-dark cycles entrained the daily rhythms of oviposition and courtship in female medaka (91). Tissue distributions have been reported for the four receptor subtypes in several teleosts (7, 10, 15, 92, 93). In teleost fish, studies by semi-quantitative RT-PCR showed that the four melatonin receptors are widely expressed and show overlapping expression patterns. In goldfish, one receptor of each vertebrate MTNR subtype has been cloned (8), the presence of other *mtnr* duplicates resulting to 3R and to the specific cyprinid genome tetraploidization 4R has not been reported (8, 37). The four paralogs, identified in this present study as *mtnr1aa*α *(named Mel1a17, LOC113044183), mtnr1bb*α *(named Mel1b, LOC113095390 and LOC113071669) mtnr1ca*α (named *Mel1c*, LOC113048834), and *mtnr1d*α *(named Mel1a14, LOC113114534)* are found in most of the areas of the brain, retina and pituitary as well as in peripheral tissues such as gill, skin, liver, intestine, kidney, scale, and spleen (8). In vertebrates, diurnal variation of *mtnr* expression, depends on the tissue and photoperiodic regime (8, 10, 11, 94–96). In Atlantic salmon, the daily expression of two of *mtnr1a* paralogs and the single *mtnr1b* increases during the spring, and adopts a parallel daily sinusoidal expression pattern with higher level of expression during the night and the early morning (10).

Our results suggest that the four melatonin receptors mediate distinct central and peripheral actions of melatonin in medaka. As in a previous study in medaka, high expression of *mtnr1a, mtnr1b*, and *mtnr1d* paralogs occurred in the brain and eye, which concurs with the role of the brain in the integration of photoperiodic signal (11, 14, 97, 98).

Interestingly, expression of *mtnr1a* and *mtnr1c* was found also in the ovary. The expression of *mtnr1a* in ovary in gar and bowfin indicates that the pre-3R ovarian function might be conserved in medaka *mtnr1a*. In contrast, *mtnr1c* was barely detectable in the ovary in other teleosts, suggesting neofunctionalization of this paralog in medaka. Melatonin can exert a direct control on gonad development and steroidogenesis, supporting the view that melatonin is a regulator of fish oocyte growth and maturation in fish (99). This role of melatonin is supported by the observation that photoperiod affects both oocyte development and egg quality in teleosts (99–101). In hens, melatonin is assumed to promote ovulation after binding to MTNR1B through down-regulation of gonadotropin-inhibitory hormone receptor (GnIHR) and stimulation of 17β-estradiol production (102).

Our study reveals high levels of *mtnr1b* mRNA in medaka adipose tissue, which surprisingly increased during the light phase. Interestingly, melatonin administration prevents visceral fat deposition in diet-induced obese zebrafish (103). In mammals, melatonin controls the brown adipose content (104, 105). Both MTNR1A (MT1) and MTNR1B (MT2) are present in human brown tissue adypocytes indicating a possible involvement of MTNR1B in adipocyte physiology (106). A study investigating mechanisms regulating hibernation in European hamster (*Cricetus cricetus*) reported altered gene expression of *MTNR1B* (named MT2) in brown adipose tissue during the hibernation cycle (107). Daily rhythmicity with higher expression during the day were observed for *mtnr1a* and *mtnr1d* in the liver of rabbitfish (*Signatus guttatus*) which is involved in the maintenance of lipid homeostasis (92, 93). These results raise new questions about the role of melatonin in modulating daily and seasonal fat storage in fish.

Melatonin plays a critical role in major anti-aging-related cardiovascular diseases including heart failure [for review see (108, 109)]. In rat both MTNR1A (MT1) and MTNR1B (MT2) are expressed in myocardium, and MTNR1B was upregulated after myocardial ischemia/reperfusion in mice (110). Both *mtnr1a* and *mtnr1c* showed changes in transcript levels in the heart during the day in medaka, suggesting that photoperiod manipulation may affect heart activity in fish.

The expression levels of three of the four melatonin receptors changed between day and night in medaka. We found different sets of *mtnr* genes expressed in the integument of teleost species: *mtnr1b* and *mtnr1d* in sole (*Solea senegalensis*) (94), *mtnr1a* and *mtnr1d* in mudskipper (*Boleophthalmus pectinirostris)* (95), *mtnr1aa*α, *mtnr1aa*β, *mtnr1ab*, and *mtnt1b* in Atlantic salmon (10) and *mtnr1b, mtnr1c*, and *mtnr1d* in goldfish (8). Melatonin induces hypopigmentation in several ostariophysian species, by stimulating aggregation of melanophores partially through stimulation of Mtnr1a and Mtnr1b (111–113). In mammals, both *MTNR1A* and *MTNR1B* are expressed in the skin with different distributions depending of the species [for review see (114)]. Melatonin exerts different functions in the skin of mammals such as pigmentation, hair growth, thermoregulation, and anticancer activity [for review see (114)], but little is known about the role of MTNR signaling in the integument physiology.

The larger number of transcripts retrieved from the PhyloFish database belonging to the MTNR1A subtype suggests that melatonin exerts its action by activating preponderantly the MTNR1A pathway. With the ayu *mtnr1b*, the PhyloFish database provided only a single example of functional divergence of 3R-paralogs. The medaka *mtnr1b* expression profile was comparable to that of the ayu *mtnr1bb*, suggesting that the 3R-paralog functional divergence began before the loss of *mtnr1ba* in medaka. The analysis of the *mtnr* from the PhyloFish database, reveals variable distribution profiles even between 3R orthologs.

Previous studies have shown that *mtnr* gene expression varies depending on the time of day and photoperiod (10). It shows the importance of considering the photoperiod regime and the time of day when comparing expression profiles of *mtnr* and other daily and seasonal oscillator genes.

In conclusion, our study provides a comprehensive overview of the evolution of melatonin receptor genes and their functional diversification in the main vertebrate taxa. The wide distribution of melatonin receptors illustrates the multi-organ action of melatonin. As in tetrapods, the four paralogs are conserved in medaka, which thereby constitutes a good model for studying melatonin function in both ecological and medical research.

## Data Availability Statement

All the data used in the articles are from the public database. All the references are provided in the tables. Alignments and the PhyML files are available in https://figshare.com/s/2dfc1acecff54f6cb1ea.

## Ethics Statement

The animal study was reviewed and approved by the Norwegian Animal Health Authority and of the Norwegian University of Life Sciences.

## Author Contributions

GM conceived the study, designed and performed the experiments, analyzed the data, wrote and revised the manuscript. RN-L performed the set up and the analysis of the qPCR experiments. F-AW conceived the study, designed the experiments, and revised the manuscript. All authors approved the final version of the manuscript.

## Conflict of Interest

The authors declare that the research was conducted in the absence of any commercial or financial relationships that could be construed as a potential conflict of interest.

## Supplementary Material

Supplementary Table 1 References of melatonin-receptor sequences used for phylogenetic analysis. The table provides the species names, gene prediction/annotation, names, identifiers, and locus references.

Supplementary Table 2 References and location of the genes used for the melatonin-receptor synteny analysis. The tables list the neighboring genes of the melatonin receptors in human, platypus, chicken, lizard, frog, shark, spotted gar, eel, arowana, herring, zebrafish, pike, cod, and medaka genome assemblies. The data includes gene name, identifier, and location. The table pre-1R lists the neighboring genes in human and in pre-vertebrate genome reconstruction (29).

Supplementary Table 3 Primer sets used for quantitative PCR. The table provides the name and 5'-3' nucleotide sequences, the efficiency, as well as the mean Cq in the brain, of the primer sets used for qPCR of the four melatonin receptors and reference genes in medaka.

Supplementary Figure 1 Tissue distribution of reference genes. Gene expression profiles of three reference genes, *18s, gapdh*, and *rpl7*, in brain (Br), pituitary (Pit), eye (Ey), gill (Gi), heart (He), spleen (Sp), adipose tissue (AT), liver (Li), intestine (Int), muscle (Mu), skin (Sk), kidney (Ki), and ovary (Ov) of reproductively active medaka females. The bar plots represent relative expression calculated as the percentage of the mean (mean ± se) per tissue.

Supplementary Figure 2 Maximum-likelihood phylogeny tree of melatonin receptor in vertebrates. Melatonin receptor phylogeny was inferred from alignment of deduced amino-acid sequences of melatonin receptor genes using the PhyML algorithm with the AIC selection criteria of the Smart Model Selection and the tree Subtree Pruning and Regrafting (SPR) improvement algorithm. The echinoderm and cephalochordate melatonin-like receptors (*mtnr-like*) were used to root the tree. Branch nodes are supported by bootstrap analysis with 100 replicates. Sequence references are provided in Supplementary Table 2.

Supplementary Figure 3 Maximum-likelihood phylogeny tree of melatonin receptor A (*mtnr1a*). Melatonin receptor phylogeny was inferred from the alignment of melatonin receptor A coding nucleotide sequences (*mtnr1a*) using the PhyML algorithm, with the AIC selection criteria of the Smart Model Selection and the tree Subtree Pruning and Regrafting (SPR) improvement algorithm. Branch nodes are supported by bootstrap analysis with 100 replicates. Asterisks (^*^) indicate partial gene sequences; PS indicate pseudogenes. Sequence references are provided in Supplementary Table 2.

Supplementary Figure 4 Maximum-likelihood phylogeny tree of melatonin receptor B (*mtnr1b*). Melatonin receptor phylogeny was inferred from the alignment of melatonin receptor B coding nucleotide sequences (*mtnr1b*) using the PhyML algorithm with the AIC selection criteria of Smart Model Selection and the SPR improvement algorithm. Branch nodes are supported by bootstrap analysis with 100 replicates. Asterisks (^*^) indicate partial gene sequences; PS indicate pseudogenes. Sequence references are provided in Supplementary Table 2.

Supplementary Figure 5 Maximum-likelihood phylogeny tree of melatonin receptor C (*mtnr1c*). Melatonin receptor phylogeny was inferred from the alignment of melatonin receptor C coding nucleotide sequences (*mtnr1c*) using the PhyML algorithm with the AIC selection criteria of Smart Model Selection and SPR improvement algorithm. Branch nodes are supported by bootstrap analysis with 100 replicates. Asterisks (^*^) indicate partial gene sequences; PS indicate pseudogenes. Sequence references are provided in Supplementary Table 2.

Supplementary Figure 6 Maximum-likelihood phylogeny tree of melatonin receptor D (*mtnr1d*). Melatonin receptor phylogeny was inferred from the alignment of melatonin receptor D (A-like) coding nucleotide sequences (*mtnr1d (al)*) using the PhyML algorithm with the AIC selection criteria of Smart Model Selection and the SPR improvement algorithm. Branch nodes are supported by bootstrap analysis with 100 replicates. Asterisks (^*^) indicate partial gene sequences; PS indicate pseudogenes. Sequence references are provided in Supplementary Table 2.

Supplementary Figure 7 Venn diagram showing the distribution of gene family members on the mtnr-bearing chromosomes in the spotted gar **(A)** and human **(B)** genomes.

Supplementary Figure 8 Maximum-likelihood phylogeny tree of melatonin receptor in vertebrates and related melatonin receptors in non-vertebrates. Melatonin receptor phylogeny was inferred from alignment of the deduced amino-acid sequences of vertebrate melatonin receptor (V-MTNR1) and non-vertebrate melatonin receptor-like (NV-MTNR-like) genes. Sequences of ospin receptors (OPN), adrenoceptor beta (ADRB), lysophosphatidic acid receptor (LPAR), melanocortin receptors (MCR) and adenosine A receptors (ADORA) and G protein-coupled receptor 52 and 21 were included to assess the relationships of the NV-MTNR-like with the V-MTNR1. Alignment was curated using Gblock and the tree was built using the PhyML algorithm with the AIC selection criteria of Smart Model Selection and the tree Subtree Pruning and Regrafting (SPR) improvement algorithm. Branch nodes are supported by bootstrap analysis with 100 replicates. Sequence references are provided in Supplementary Table 2.

Supplementary Figure 9 Evolution of the chromosomes carrying the *mtnr* genes in Clupeocephala. A. Comparison of the chromosomal distribution of *mtnr* in three clupeocephalan species including the denticle herring, the Northern pike and the Japanese medaka (HdrR). The color in the chromosomes indicates region homologous with chromosomes carrying the *mtnr* in the spotted gar (used as reference genome for the pre-3R). The figure was adapted from the multi-genome comparison between the spotted gar and three clupeocephalan species performed on the Genomicus browser. B. Evolutionary scenario of the chromosomes carrying the *mtnr* in the ancestor of the Clupeocephala. Our data showed that paralogous genomic regions carrying the *mtnr1c* and the *mtnr1d* were combined into the same chromosome during the intense genome rearrangement events that took place prior the teleost tetraploidization (3R). The 3R paralog of the *mtnr1d* gene co-localizing with the *mtnr1cb* paralog, was lost shorty after the 3R and before the teleost radiation. Further genomic inter-chromosomal rearrangements occurred in the ancestor of the Clupeocephala that lead to the linkage of the genomic region of *mtnr1ab* with the one of *mtnr1ca* and *mtnr1d* pair, and to the linkage of the genomic regions of *mtnr1bb* and *mtnr1cb*.
