# Corrigendum: New Insights Into the Evolutionary History of Melatonin Receptors in Vertebrates, With Particular Focus on Teleosts

**DOI:** 10.3389/fendo.2020.610274

**Published:** 2020-10-28

**Authors:** Gersende Maugars, Rasoul Nourizadeh-Lillabadi, Finn-Arne Weltzien

**Affiliations:** Physiology Unit, Faculty of Veterinary Medicine, Norwegian University of Life Sciences, Oslo, Norway

**Keywords:** melatonin receptors, gene duplication, vertebrates, teleosts, medaka, phylogeny, synteny, functional evolution

In the original article, there was a mistake in the **Figure 9** as published. In the Figure, we have represented the *mtnr1ba* gene in the herring while it has been lost in this lineage. The corrected **Figure 9** appears below.

**Figure 9:**
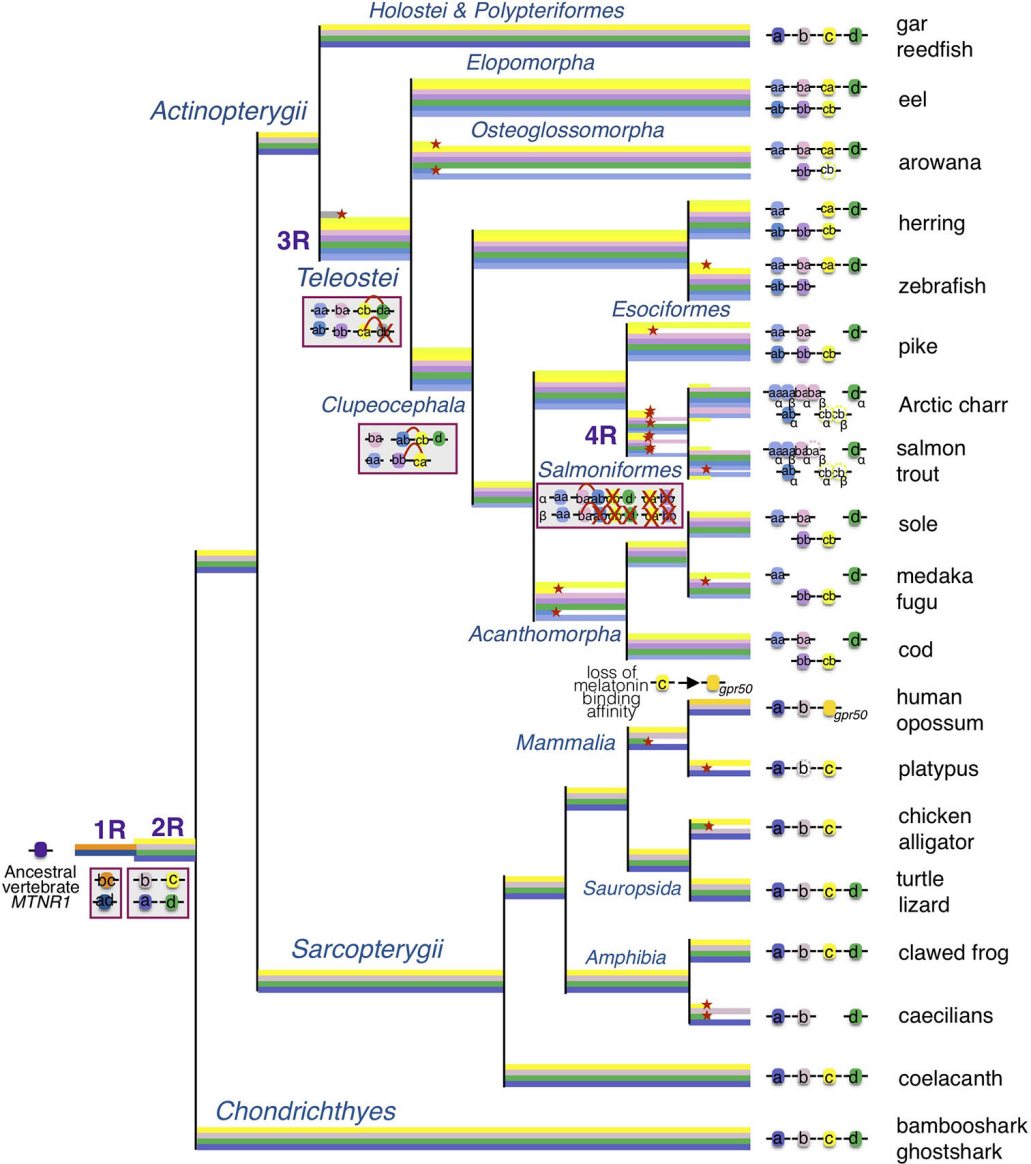
Evolutionary scenario of the melatonin receptors in vertebrates. This evolutionary scenario was developed on the basis of the phylogeny and synteny analyses presented in the original article. The four receptor genes are derived from duplication of an ancient *mtnr1* gene through vertebrate tetraploidization (1R and 2R). The teleost 3R event generated duplicates of the four *mtnr* subtypes. Multiple and selective gene losses occurred, leading to *mtnr* gene repertoires differing between the main gnathostome lineages. Genome tetraploidization events (1R, 2R, 3R, and 4R) are indicated in purple. Major gene gain and loss events as well as chromosome rearrangements are indicated in red boxes. Genes located on the same linkage group are represented by clusters on the genomic DNA line. Red cross indicates gene loss. The red arc indicates genomic region fusion events. Colored tree branches represent *mtnr* gene lineages. Red star * on a tree branch indicates gene loss. The receptor identities are indicated in or beside the boxes. Paralogs originating from teleost 3R are designated by a and b suffixes, and salmonid 4R paralogs are designated by α and β suffixes.

The authors apologize for this error and state that this does not change the scientific conclusions of the article in any way. The original article has been updated.

