# Supplementary figures and images for: New Insights Into the Evolutionary History of Melatonin Receptors in Vertebrates, With Particular Focus on Teleosts
Source: Front Endocrinol (Lausanne). 2020 Sep 24;11:538196. doi: 10.3389/fendo.2020.538196 (PMC7541902; doi:10.3389/fendo.2020.538196)

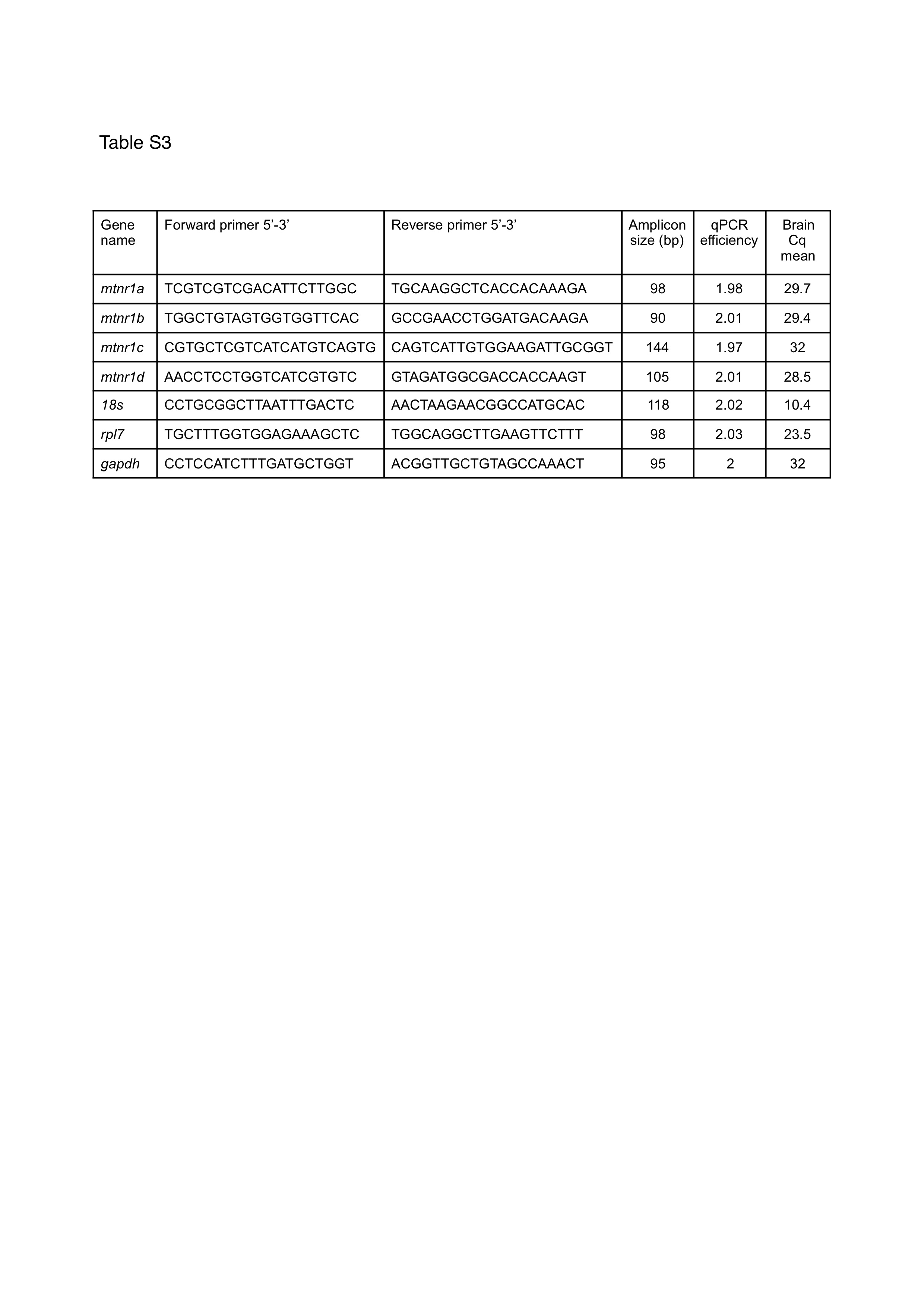

Supplement: Supplementary Table 3 — Primer sets used for quantitative PCR. The table provides the name and 5'-3' nucleotide sequences, the efficiency, as well as the mean Cq in the brain, of the primer sets used for qPCR of the four melatonin receptors and reference genes in medaka. [file Image_10.JPEG]

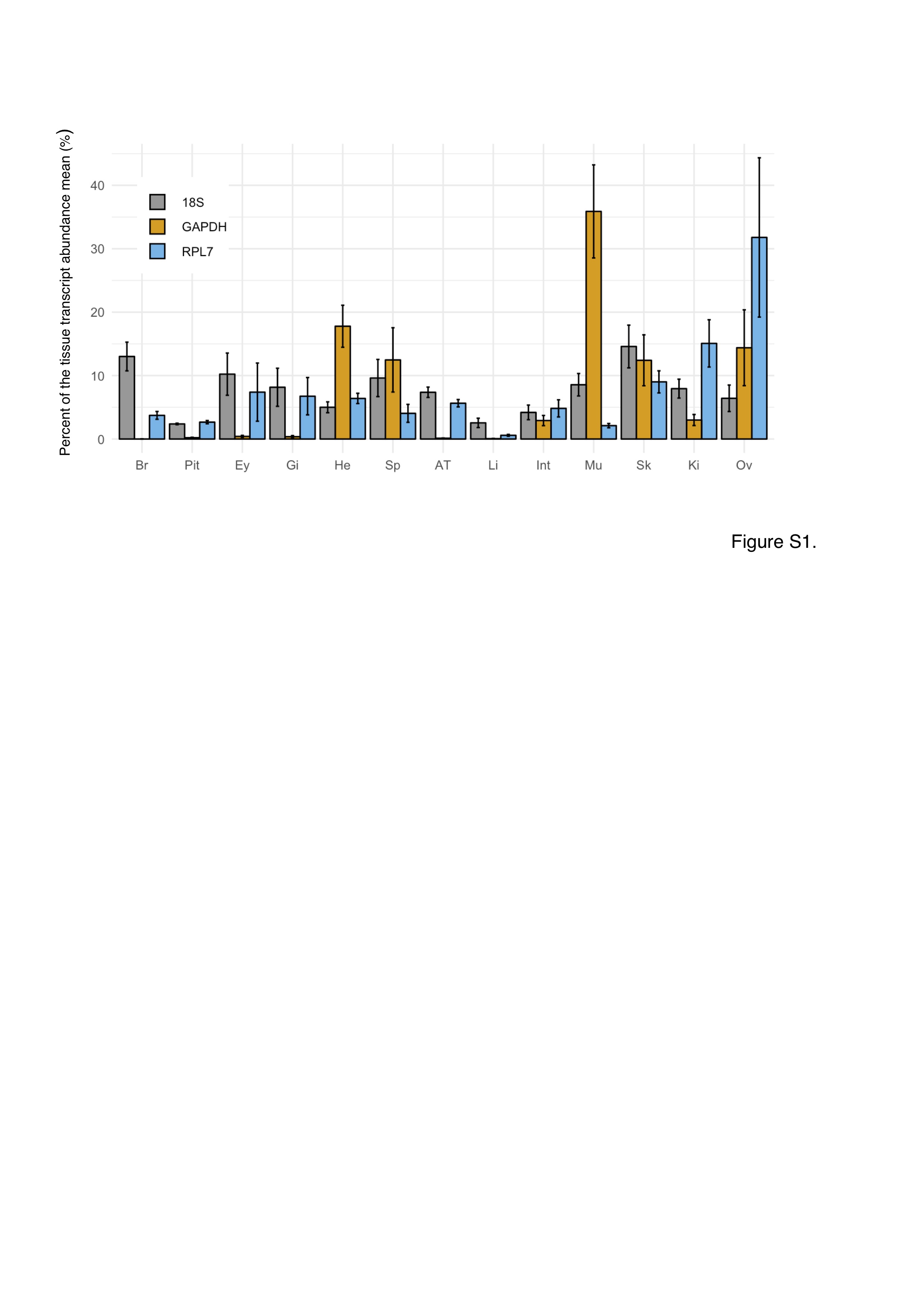

Supplement: Supplementary Figure 1 — Tissue distribution of reference genes. Gene expression profiles of three reference genes, 18s, gapdh, and rpl7, in brain (Br), pituitary (Pit), eye (Ey), gill (Gi), heart (He), spleen (Sp), adipose tissue (AT), liver (Li), intestine (Int), muscle (Mu), skin (Sk), kidney (Ki), and ovary (Ov) of reproductively active medaka females. The bar plots represent relative expression calculated as the percentage of the mean (mean ± se) per tissue. [file Image_1.JPEG]

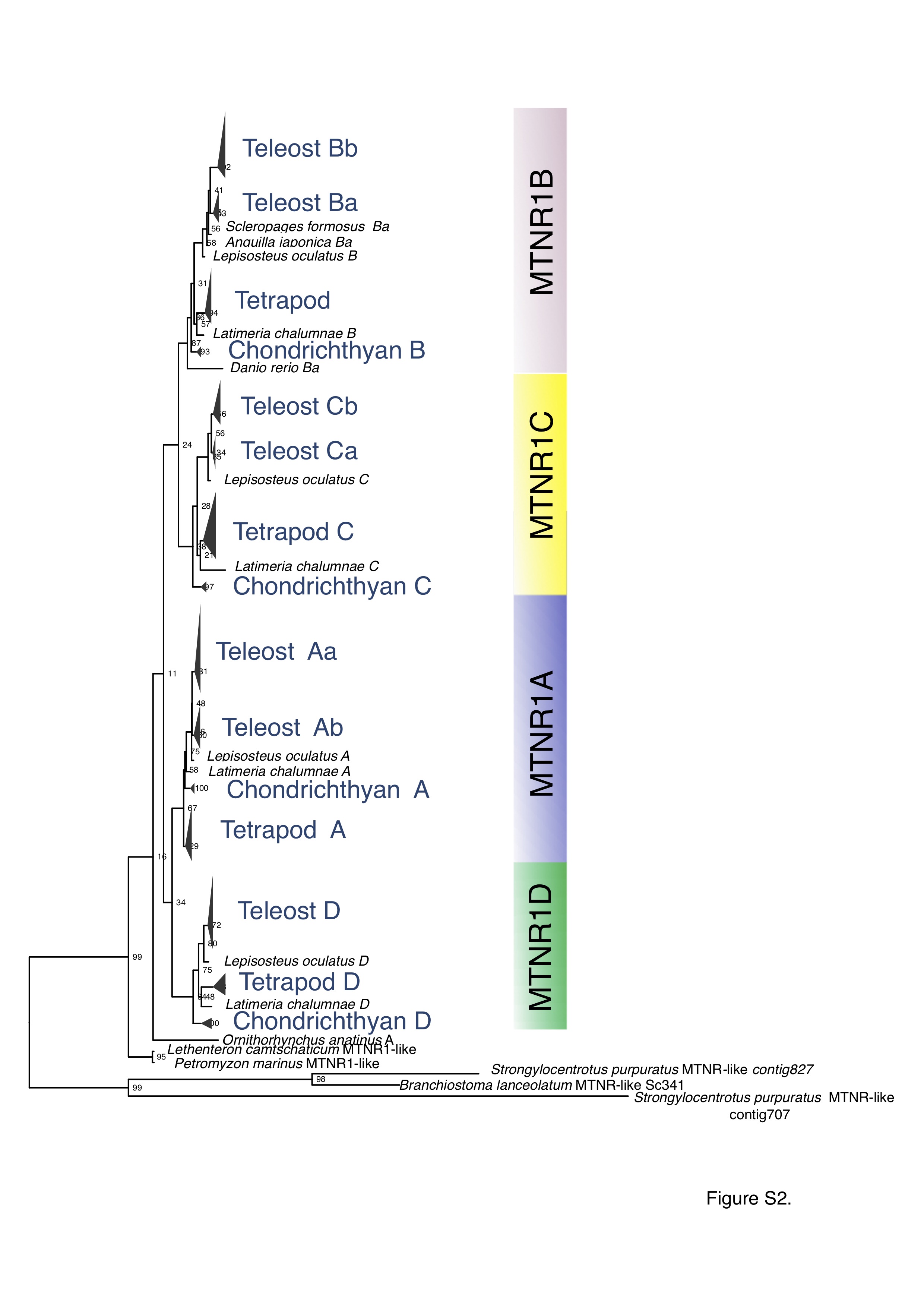

Supplement: Supplementary Figure 2 — Maximum-likelihood phylogeny tree of melatonin receptor in vertebrates. Melatonin receptor phylogeny was inferred from alignment of deduced amino-acid sequences of melatonin receptor genes using the PhyML algorithm with the AIC selection criteria of the Smart Model Selection and the tree Subtree Pruning and Regrafting (SPR) improvement algorithm. The echinoderm and cephalochordate melatonin-like receptors (mtnr-like) were used to root the tree. Branch nodes are supported by bootstrap analysis with 100 replicates. Sequence references are provided in Supplementary Table 2. [file Image_2.JPEG]

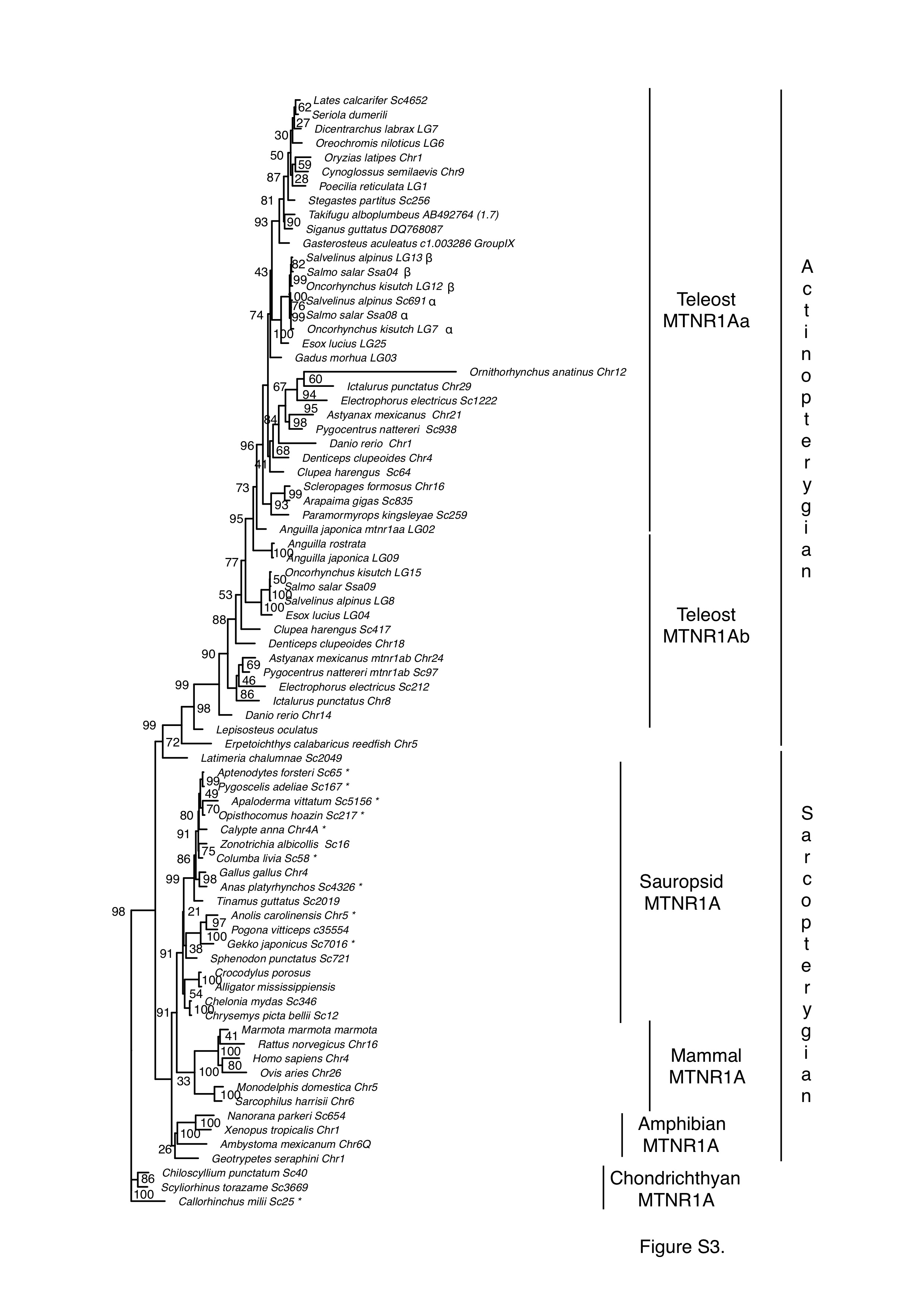

Supplement: Supplementary Figure 3 — Maximum-likelihood phylogeny tree of melatonin receptor A (mtnr1a). Melatonin receptor phylogeny was inferred from the alignment of melatonin receptor A coding nucleotide sequences (mtnr1a) using the PhyML algorithm, with the AIC selection criteria of the Smart Model Selection and the tree Subtree Pruning and Regrafting (SPR) improvement algorithm. Branch nodes are supported by bootstrap analysis with 100 replicates. Asterisks (*) indicate partial gene sequences; PS indicate pseudogenes. Sequence references are provided in Supplementary Table 2. [file Image_3.JPEG]

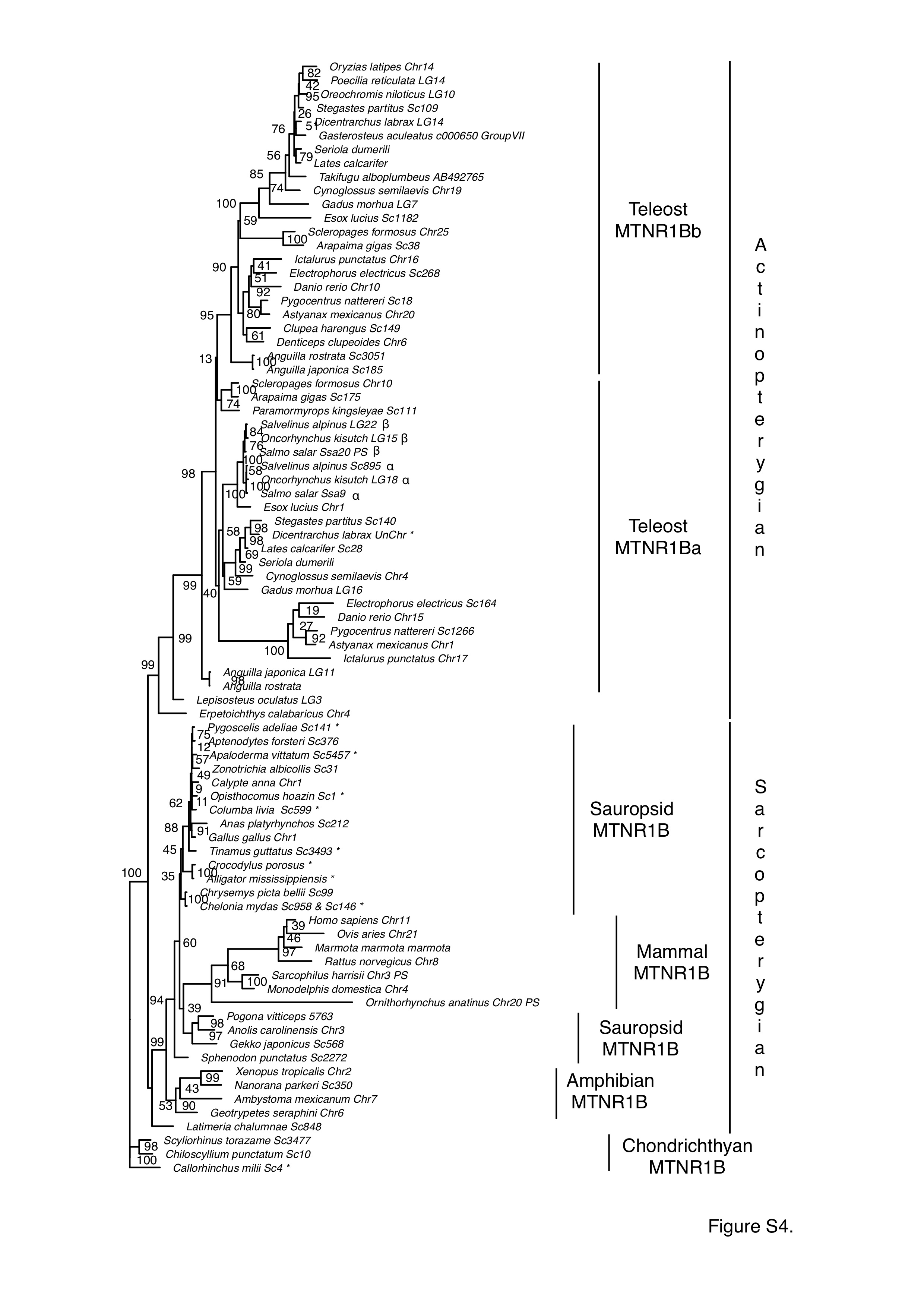

Supplement: Supplementary Figure 4 — Maximum-likelihood phylogeny tree of melatonin receptor B (mtnr1b). Melatonin receptor phylogeny was inferred from the alignment of melatonin receptor B coding nucleotide sequences (mtnr1b) using the PhyML algorithm with the AIC selection criteria of Smart Model Selection and the SPR improvement algorithm. Branch nodes are supported by bootstrap analysis with 100 replicates. Asterisks (*) indicate partial gene sequences; PS indicate pseudogenes. Sequence references are provided in Supplementary Table 2. [file Image_4.JPEG]

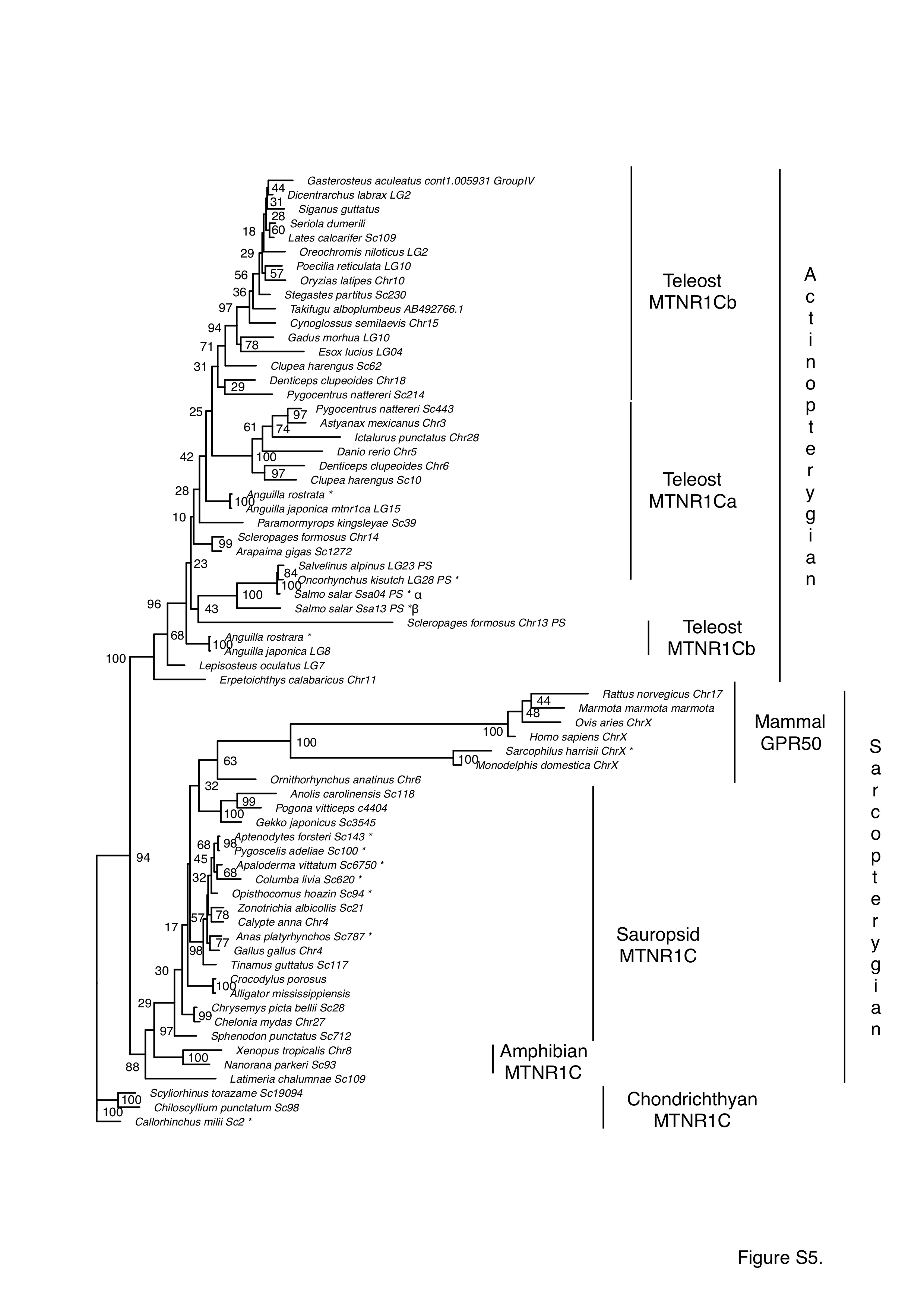

Supplement: Supplementary Figure 5 — Maximum-likelihood phylogeny tree of melatonin receptor C (mtnr1c). Melatonin receptor phylogeny was inferred from the alignment of melatonin receptor C coding nucleotide sequences (mtnr1c) using the PhyML algorithm with the AIC selection criteria of Smart Model Selection and SPR improvement algorithm. Branch nodes are supported by bootstrap analysis with 100 replicates. Asterisks (*) indicate partial gene sequences; PS indicate pseudogenes. Sequence references are provided in Supplementary Table 2. [file Image_5.JPEG]

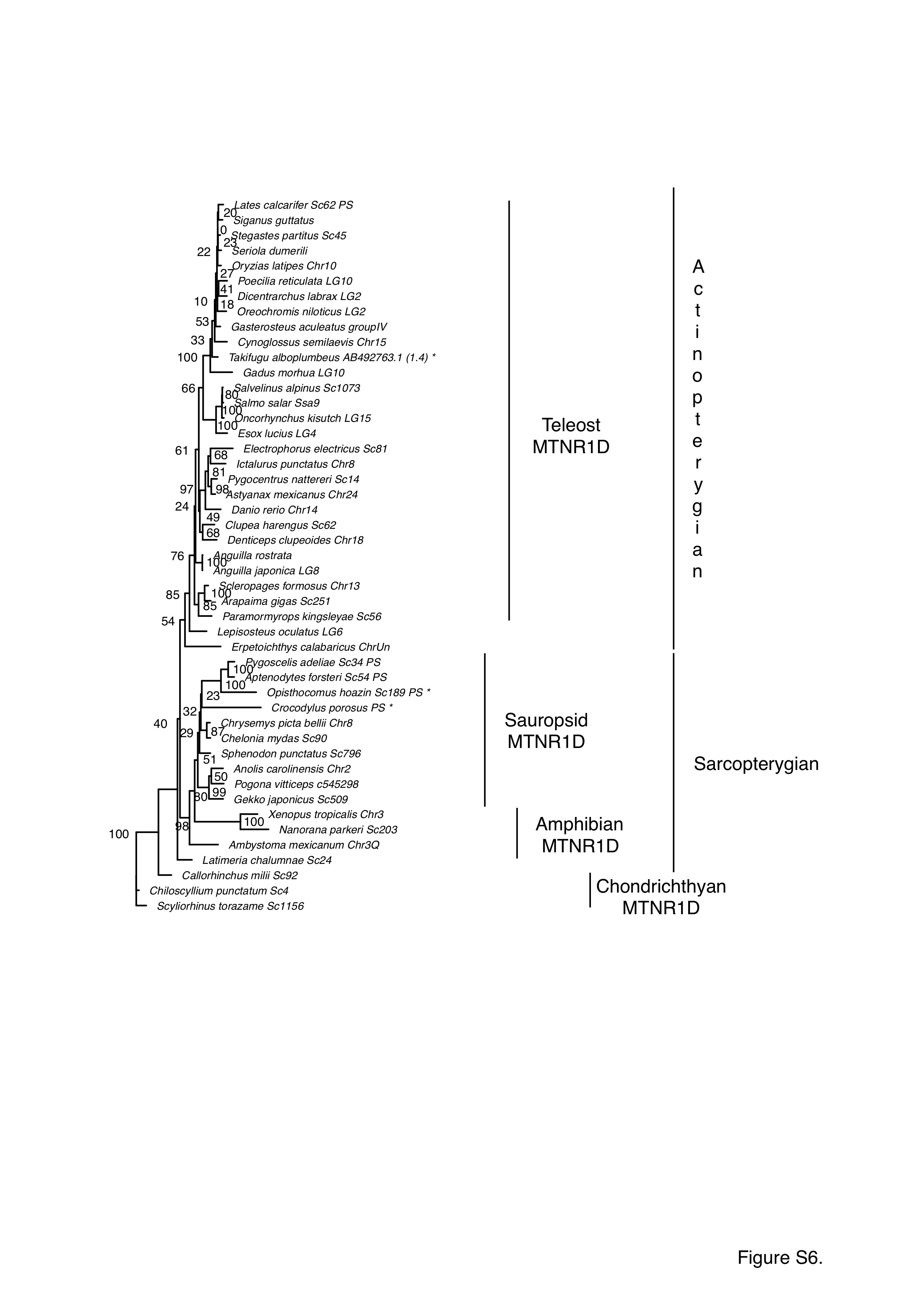

Supplement: Supplementary Figure 6 — Maximum-likelihood phylogeny tree of melatonin receptor D (mtnr1d). Melatonin receptor phylogeny was inferred from the alignment of melatonin receptor D (A-like) coding nucleotide sequences (mtnr1d (al)) using the PhyML algorithm with the AIC selection criteria of Smart Model Selection and the SPR improvement algorithm. Branch nodes are supported by bootstrap analysis with 100 replicates. Asterisks (*) indicate partial gene sequences; PS indicate pseudogenes. Sequence references are provided in Supplementary Table 2. [file Image_6.JPEG]

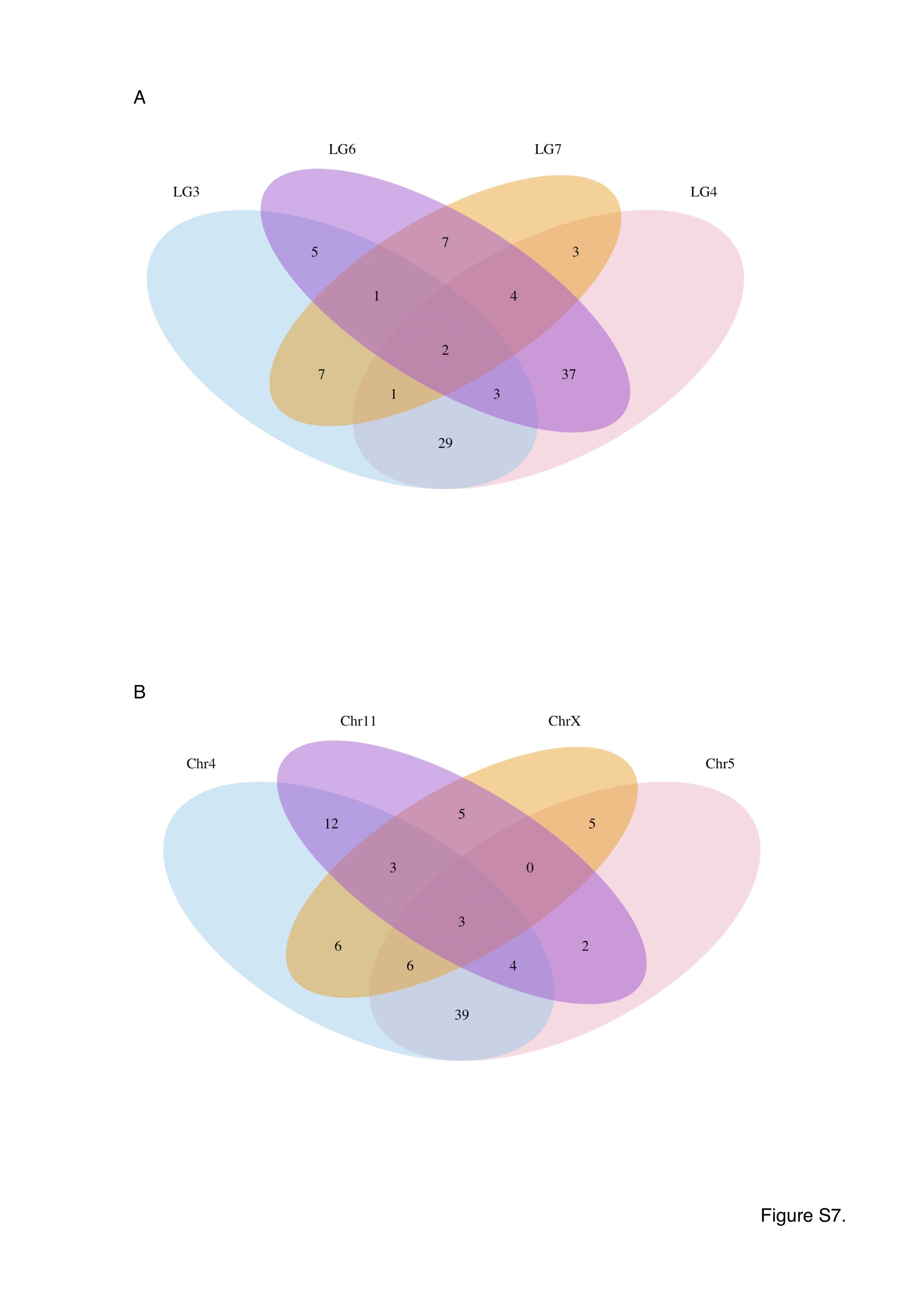

Supplement: Supplementary Figure 7 — Venn diagram showing the distribution of gene family members on the mtnr-bearing chromosomes in the spotted gar (A) and human (B) genomes. [file Image_7.JPEG]

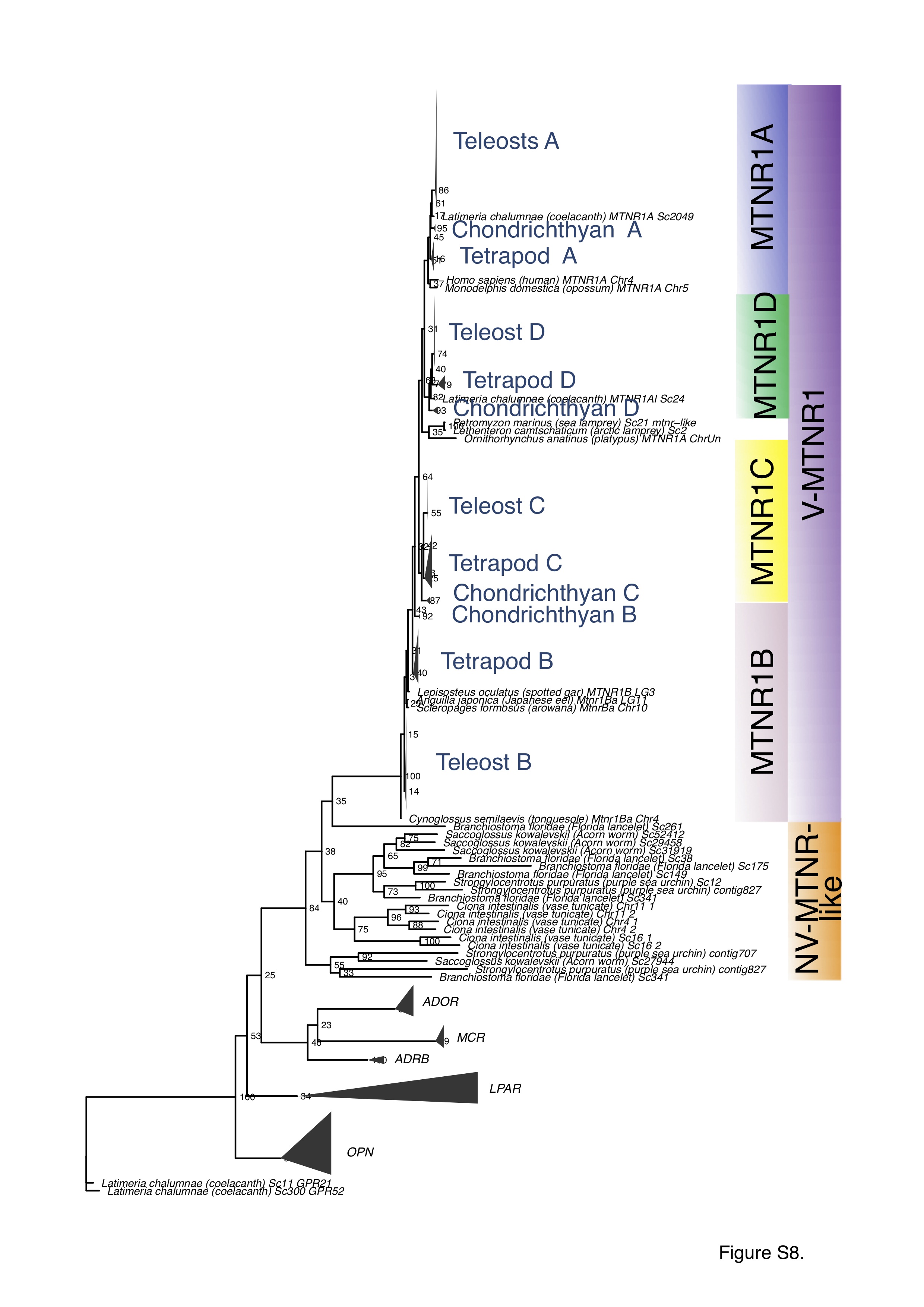

Supplement: Supplementary Figure 8 — Maximum-likelihood phylogeny tree of melatonin receptor in vertebrates and related melatonin receptors in non-vertebrates. Melatonin receptor phylogeny was inferred from alignment of the deduced amino-acid sequences of vertebrate melatonin receptor (V-MTNR1) and non-vertebrate melatonin receptor-like (NV-MTNR-like) genes. Sequences of ospin receptors (OPN), adrenoceptor beta (ADRB), lysophosphatidic acid receptor (LPAR), melanocortin receptors (MCR) and adenosine A receptors (ADORA) and G protein-coupled receptor 52 and 21 were included to assess the relationships of the NV-MTNR-like with the V-MTNR1. Alignment was curated using Gblock and the tree was built using the PhyML algorithm with the AIC selection criteria of Smart Model Selection and the tree Subtree Pruning and Regrafting (SPR) improvement algorithm. Branch nodes are supported by bootstrap analysis with 100 replicates. Sequence references are provided in Supplementary Table 2. [file Image_8.JPEG]

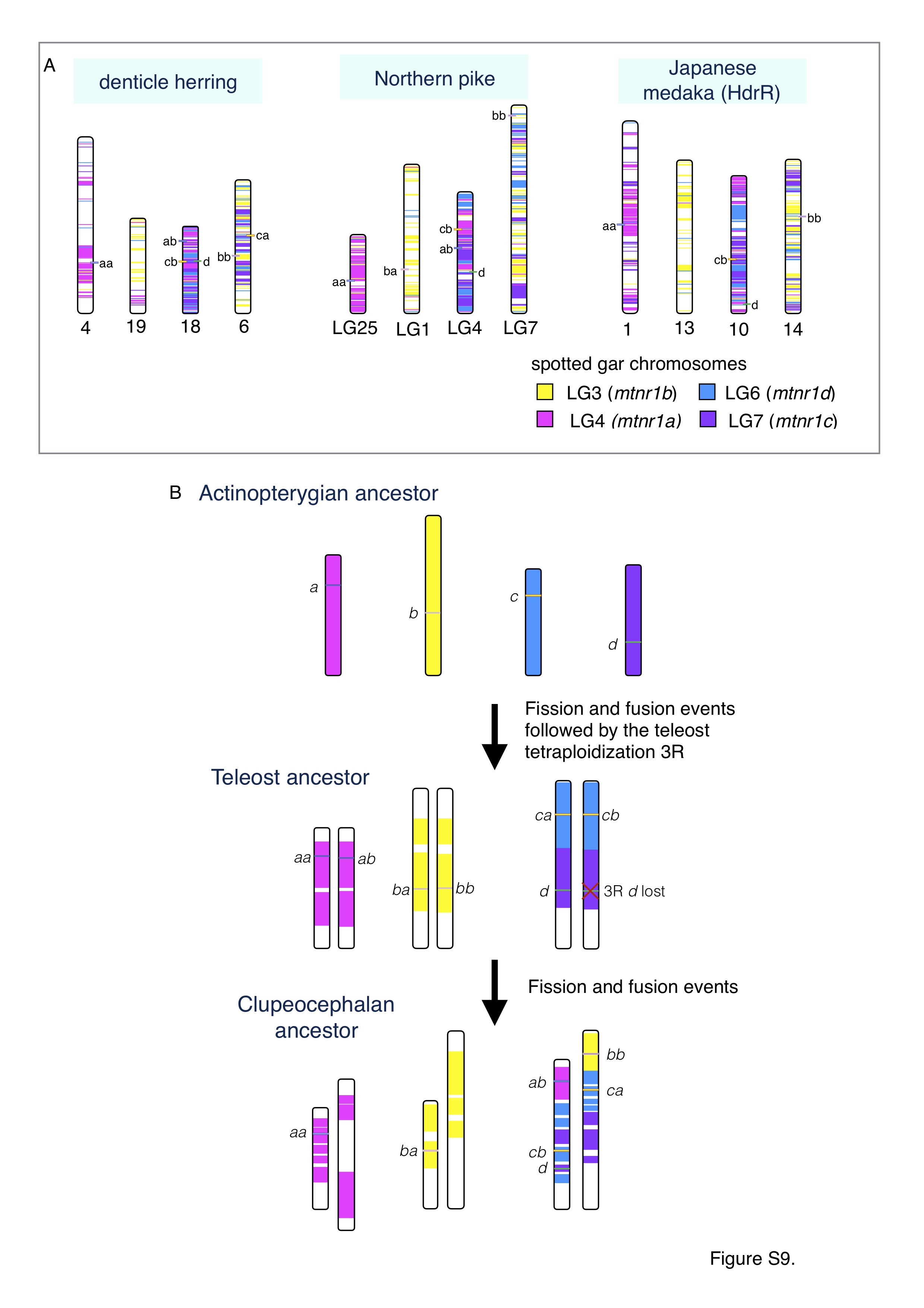

Supplement: Supplementary Figure 9 — Evolution of the chromosomes carrying the mtnr genes in Clupeocephala. A. Comparison of the chromosomal distribution of mtnr in three clupeocephalan species including the denticle herring, the Northern pike and the Japanese medaka (HdrR). The color in the chromosomes indicates region homologous with chromosomes carrying the mtnr in the spotted gar (used as reference genome for the pre-3R). The figure was adapted from the multi-genome comparison between the spotted gar and three clupeocephalan species performed on the Genomicus browser. B. Evolutionary scenario of the chromosomes carrying the mtnr in the ancestor of the Clupeocephala. Our data showed that paralogous genomic regions carrying the mtnr1c and the mtnr1d were combined into the same chromosome during the intense genome rearrangement events that took place prior the teleost tetraploidization (3R). The 3R paralog of the mtnr1d gene co-localizing with the mtnr1cb paralog, was lost shorty after the 3R and before the teleost radiation. Further genomic inter-chromosomal rearrangements occurred in the ancestor of the Clupeocephala that lead to the linkage of the genomic region of mtnr1ab with the one of mtnr1ca and mtnr1d pair, and to the linkage of the genomic regions of mtnr1bb and mtnr1cb. [file Image_9.jpg]
